# Is There a Role for GPCR Agonist Radiotracers in PET Neuroimaging?

**DOI:** 10.3389/fnmol.2019.00255

**Published:** 2019-10-18

**Authors:** Matthieu Colom, Benjamin Vidal, Luc Zimmer

**Affiliations:** ^1^Lyon Neuroscience Research Center, INSERM, CNRS, Université de Lyon, Lyon, France; ^2^CERMEP, Hospices Civils de Lyon, Bron, France; ^3^Institut National des Sciences et Techniques Nucléaires, CEA Saclay, Gif-sur-Yvette, France

**Keywords:** radiopharmaceutical, receptor, agonist, Positron emission tomography (PET), neuroimaging

## Abstract

Positron emission tomography (PET) is a molecular imaging modality that enables *in vivo* exploration of metabolic processes and especially the pharmacology of neuroreceptors. G protein-coupled receptors (GPCRs) play an important role in numerous pathophysiologic disorders of the central nervous system. Thus, they are targets of choice in PET imaging to bring proof concept of change in density in pathological conditions or in pharmacological challenge. At present, most radiotracers are antagonist ligands. *In vitro* data suggest that properties differ between GPCR agonists and antagonists: antagonists bind to receptors with a single affinity, whereas agonists are characterized by two different affinities: high affinity for receptors that undergo functional coupling to G-proteins, and low affinity for those that are not coupled. In this context, agonist radiotracers may be useful tools to give functional images of GPCRs in the brain, with high sensitivity to neurotransmitter release. Here, we review all existing PET radiotracers used from animals to humans and their role for understanding the ligand-receptor paradigm of GPCR in comparison with corresponding antagonist radiotracers.

## Introduction

### From Agonist Molecules to PET Radiopharmaceuticals

Positron emission tomography is a molecular imaging modality that enables exploration of metabolic processes *in vivo*. It uses specific radiotracers for specific molecular targets (Van de Bittner et al., 2014). The radiotracer must have several characteristics (Halldin et al., 2001; Pike, 2009; Honer et al., 2014): i.e., a specific binding to the target of interest with an acceptable signal-to-noise ratio, a passage through the BBB, and limited radiometabolites in the brain. The most common molecular targets in PET neuroimaging are neurotransmitter receptors or transporters. PET imaging visualizes various neuroreceptors that can be located *in vivo* on presynaptic and/or post-synaptic sites, using a microdose of radioligand (i.e., tracer dose). PET imaging is therefore a powerful tool to demonstrate changes in neurotransmission in various CNS disorders, and can be used translationally in both animal models and humans. In addition to its contribution to the understanding of pathophysiological processes, PET imaging plays an important role in CNS drug development. It enables measurement of the proportion of receptors occupied by pharmacological doses of drugs of interest, in competition with a suitable radioligand specific to the same target. It can thus demonstrate brain penetration and *in vivo* binding to the target, which can be correlated to plasma concentrations to predict the effective dose range for clinical studies. They collect important information about the bioavailability of the drug candidate and contribute to the demonstration of brain penetration. Microdosing and drug occupancy studies have been shown to be very valuable for optimizing the development of drugs targeting the CNS. Another important application of PET neuroimaging is to measure *in vivo* fluctuations in endogenous neurotransmitter release. According to the occupancy model, the binding potential of a given radiotracer is modulated by the local concentration of the endogenous neurotransmitter in competition for the same receptors when the affinities of radioligand and neurotransmitter are in the same order of magnitude (Laruelle, 2000). All these applications rely on the development and full characterization of specific radiotracers. Consequently, although PET imaging provides interesting *in vivo* approaches to understanding neuropharmacology, it is currently limited by a lack of specific radiotracers for many known brain receptors. Moreover, the large majority of available radiotracers are antagonists and, as will be explained below, may not provide information about GPCR functional status *in vivo*; this fact, often disregarded, may be an important limitation for the interpretation of numerous clinical PET studies and probably explains certain controversies still ongoing in the field. The present review will therefore focus on the few agonist radiotracers that are currently available, highlighting their potential interest in PET neuroimaging, especially in humans in the form of radiopharmaceuticals.

### The Pharmacological Paradigm of G Protein-Coupled Receptor Agonism

**FIGURE 1:**
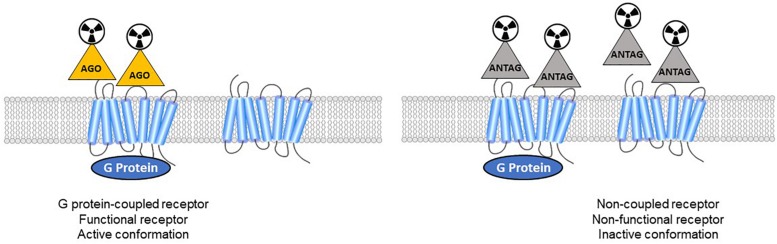
Antagonist radioligands bind both coupled and non-coupled GPCRs with the same affinity. Agonist radioligands discriminate high-affinity (active GPCRs) versus low-affinity sites (inactive receptors).

*In vitro* studies on membrane homogenates distinguished different properties in GPCR agonists and antagonists. While antagonists bind to receptors with a single affinity, agonists show two different affinities: high affinity for receptors coupled to G-proteins, and low affinity for uncoupled receptors (Figure 1). As reviewed recently (Shalgunov et al., 2019), these findings were demonstrated for various GPCRs: adrenergic (Hoffman and Lefkowitz, 1980), dopaminergic (Sibley et al., 1982; Leff et al., 1985), serotonergic (Battaglia et al., 1984; Gozlan et al., 1995; Watson et al., 2000) muscarinic (Ehlert, 1985), cannabinoid (Gullapalli et al., 2010), and opioid receptors (Law et al., 1985). The pharmacology of high-affinity receptors features specific phenomena: negative GTP cycle feedback loop, oligo-heterodimerization, internalization. While many *in vitro* studies revealed dysregulation of the balance between coupled and uncoupled states in these receptors, the implications for neurological disorders remains to be demonstrated, and new tools are needed. At present, few GPCRs can actually be studied using both antagonist and agonist radiotracers. Thus the different functional states of the receptors *in vivo* cannot be distinguished, making it impossible to specifically study the receptors in the high-affinity state which must represent the true (synapse) responsivity of the system to endogenous neurotransmission. Given that agonists bind preferentially to coupled receptors, PET imaging could disclose the active state of a receptor population.

### Antagonists Are Often Used in PET Neuroimaging

The primary reason was that radiochemists and radiopharmacologists had access to a larger choice of antagonist molecules, initially developed as neuropharmacological tools; but there are also other reasons. Firstly, in terms of pharmacology, it is simpler to use an antagonist, which has only one affinity for a given receptor, as it does not discriminate between different subpopulations but rather provides an image of the global density of receptors. In contrast, using an agonist complicates the analysis due to its dual affinity for both high- and low-affinity receptors, providing an image with lower signal-to-noise ratio due to the lower density of the targeted receptors. Moreover, agonists are more likely to induce undesirable side effects for the patient if the quantity injected is too great (i.e., higher than a tracer dose): the specific activity needs to be high enough for a microdose of agonist to be injected, at subpharmacological level. Another problem is that the rate of conversion from high- to low-affinity state can cause the agonist to dissociate from its receptor too quickly: after stimulation by the agonist, the receptor is likely to switch to the low-affinity state within seconds (Mathis et al., 1997). Although dissociation is slower than the conversion from high- to low-affinity state, this can cast doubt on the functional state of the receptors actually targeted by the agonist (Seeman, 2012). All these reasons explain why PET neuroimaging using agonist radiotracers is considered challenging, as it raises a number of difficulties relating to radiopharmacy (e.g., the potential pharmacological effects of the radioligand if the specific activity is too low), or to pharmacological concepts that are not at present fully elucidated, such as the conformational model of GPCR signaling itself. In fact, GPCRs can be considered as either pre-coupled to their respective G proteins or not (De Lean et al., 1980; Kent et al., 1980; Mongeau et al., 1992), or as initially non-coupled and interacting with G proteins after agonist stimulation (Laruelle, 2000; Skinbjerg et al., 2010).

### Agonist Radiotracers to Explore the Coupling of GPCRs

On the other hand, the fact that neuroimaging mainly uses antagonist radiotracers incurs a number of limitations, in terms of both neurophysiology and pathophysiology. For neurophysiological exploration, the lack of sensitivity to endogenous neurotransmitter level or exogenous agonists at pharmacological dose, as will be discussed below, calls for the use of agonist radioligands. The physiological impact of GPCR functional state plays a major role in maintaining cellular homeostasis and effective neurotransmission. Compared to ion channels, which produce relatively simple and quick responses after stimulation by a ligand, GPCR signaling involves complex signaling cascades with production of numerous secondary messengers, interaction with various channels (Reboreda et al., 2018) and phosphorylation of diverse proteins, which may have varying long-term effects in the cell. Therefore, GPCRs are of critical functional importance in the CNS and are one of the most important drug targets in pharmacology, and especially neuropharmacology (Albizu et al., 2010; Björk and Svenningsson, 2011; Jastrzebska et al., 2011; Hauser et al., 2017). The same GPCR can interact with multiple signaling pathways that may vary across the brain [e.g., 5-HT_1A_ receptors (la Cour, 2006)]. For example, according to the recent concept of biased agonism, each ligand may preferentially stimulate a few of the numerous pathways that can interact with a GPCR. This may explain the diverse pharmacological effects observed for a given class of GPCR ligands (Albizu et al., 2010). The complexity of GPCRs needs to be studied more extensively *in vivo*, and requires the development of suitable tools such as agonist radiotracers and, ultimately, biased agonists. Pathophysiologically, G protein signaling is strongly involved in many neurodegenerative or neuropsychiatric disorders (Schreiber and Avissar, 2000; Avissar and Schreiber, 2006; Thathiah and De Strooper, 2011; Heese, 2013). Numerous *in vitro* studies showed that GPCR coupling state is modified in pathological conditions (Schreiber et al., 2009; Becker et al., 2014; Vidal et al., 2016). The exploration of coupled and non-coupled populations of receptors *in vivo* could be a key point in developing new therapies, monitoring treatment effects and identifying treatment responders. At present, it is difficult to explore G protein coupling in the brain *in vivo* due to a lack of non-invasive tools; it is measured either on brain slices *in vitro* (Pejchal et al., 2002; Shen et al., 2002) or in peripheral cells such as leukocytes (Golan et al., 2011).

According to *in vitro* pharmacological findings, agonist radiotracers may provide new tools, challenging the standard ligand receptor model in pharmacology (Laruelle, 2000). They seem to be involved in GPCR activity and reflect the responsiveness of the synapse signaling system (Shalgunov, 2017). Therefore, agonist radioligands could provide precise *in vivo* pharmacology by imaging only active neuroreceptors (Zimmer, 2016). PET would thus seem to be the means to shed light on GPCR properties *in vivo*.

## State of the Art of Existing PET Agonist Radiotracers for Neuroimaging

**FIGURE 2:**
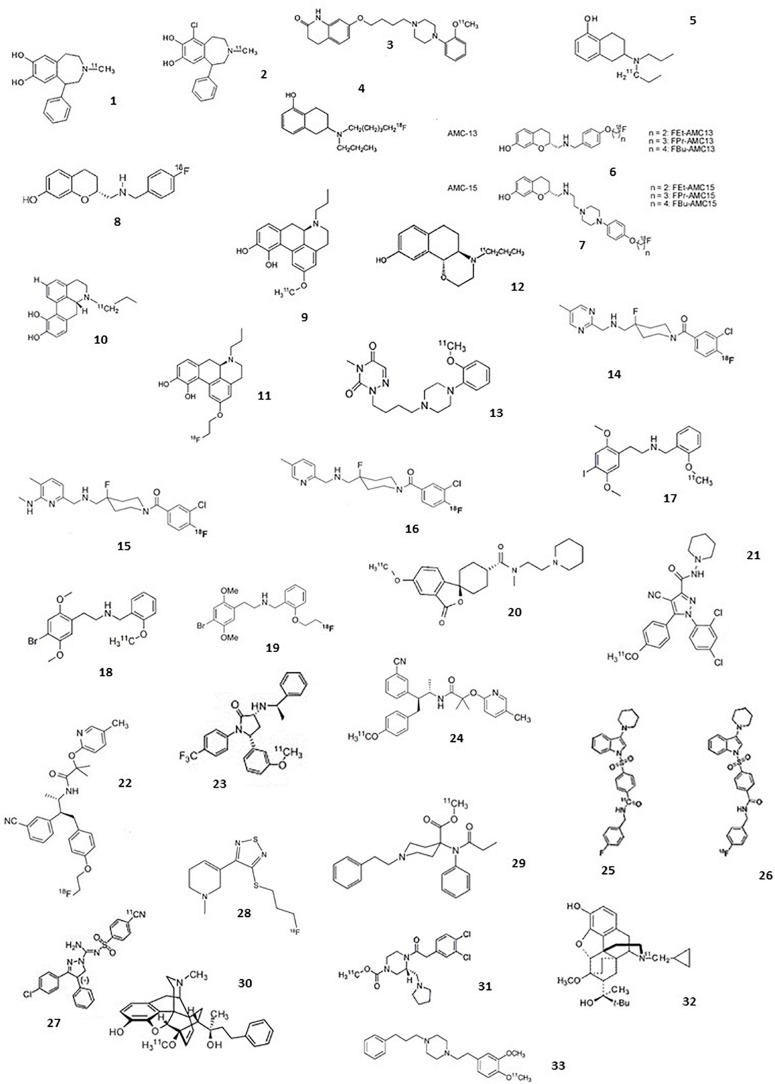
Chemical structures of current agonist radiotracers of GPCRs. Dopamine receptors: (1) [^11^C]SKF 82957, (2) [^11^C]-SKF75670, (3) [^11^C]SV-III-130, (4) [^11^C]5-OH-DPAT, (5) [^18^F]5-OH-FPPAT, (6) [^18^F]FBu-AMC13 and derivatives, (7) [^18^F]FEt-AMC15 and derivatives, (8) [^18^F]AMC20, (9) [^11^C]NPA, (10) [^11^C]MNPA, (11) [^18^F]MCL-524, (12) [^11^C]PHNO; serotonin receptors: (13) [^11^C]CUMI-101, (14) [^18^F]F15599, (15) [^18^F]F13714, (16) [^18^F]F13640, (17) [^11^C]Cimbi-5, (18) [^11^C]Cimbi-36, (19) [^18^F]FECimbi-36; histamine receptors: (20) [^11^C]MK-8278; cannabinoid receptors: (21) [^11^C]OMAR or [^11^C]JHU75528; (22) [^18^F]MK-9470, (23) [^11^C]MePPEP, (24) [^11^C]CB-119, (25) [^11^C]PipISB, (26) [^18^F]PipISB, (27) [^11^C]SD5024; acetylcholine receptors: (28) [^18^F]FP-TZTP; Opioïd receptors: (29) [^11^C]carfentanil, (30) [^11^C]PEO, (31) [^11^C]GR103545, (32) [^11^C]buprenorphine; Sigma 1 receptors: (33) [^11^C]SA4503.

The following section comprises an exhaustive review of PET radiotracers with a translation to first in human are presented (Figure 2 and Table 1). See Supplementary Material for exhaustive review.

**TABLE 1:** Current agonist radiotracers of GPCRs with at least preclinical validation.

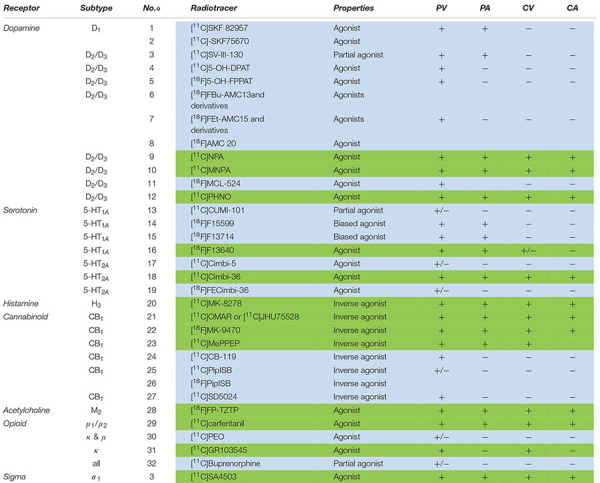

*PV, preclinical validation; PAs, preclinical applications; CV, clinical validation; CA, clinical applications. Translation to human (Green)/Only preclinical studies (Blue).*

### Dopaminergic Receptors

The dopaminergic system has five formally described subtypes of receptors (D_1_, D_2_, D_3_, D_4_, D_5_). The dopaminergic system has benefited from the development of a large number of PET radiotracers. The majority of these radiotracers are derived from the many pharmacological tools and drug candidates that have been developed for psychiatry and then neurology. Among these, the use in humans of three agonist radiotracers of D_2_/D_3_R, allowed to investigate sensitivity to neurotransmitter release and estimate the proportion of coupling in comparison with the reference antagonist radiotracer, [^11^C]raclopride.

### D_2_/D_3_ Receptors

#### [^11^C]NPA

Ernst (1967) reported that apomorphine interacted with dopamine receptors. This observation launched intensive research about apomorphine’s structure/activity relationship, to define precisely the pharmacophore enabling dopamine receptor binding/agonism/stimulation. [^11^C]apomorphine itself was synthesized and evaluated in rat brain as a radioligand of D_1_-like and D_2_-like receptors (Zijlstra et al., 1993; Finnema et al., 2010). Brain uptake and specific binding ratios were too low for satisfactory application in PET imaging. Hwang et al. (2000) proposed [^11^C] radiolabeling of NPA, first synthesized in Neumeyer et al. (1973). NPA is a D_2_ agonist with higher affinity for D_2_ receptors at high affinity than for D_2_ receptors at low affinity (Sibley et al., 1982). Previous studies demonstrated that its tritiated analog crossed the BBB, with high uptake in striatum (van der Werf et al., 1983). Hwang and colleagues described the radiosynthesis of [^11^C]NPA, and biodistribution studies in rodents confirmed high uptake in striatum and a high striatum/cerebellum ratio (3.4 at 30 min post-injection). Haloperidol pretreatment decreased the striatum/cerebellum ratio to 1.3 at 30 min in rat brain. PET imaging studies on a single baboon also revealed a high striatum/cerebellum ratio of 2.8 at 45 min post-injection. A blockade study with haloperidol strongly decreased the striatum/cerebellum ratio, confirming specific binding to D_2_/D_3_ receptors *in vivo*. Cumming et al. (2002) demonstrated that [^3^H]NPA was more sensitive to endogenous dopamine in striatum than the antagonist [^11^C]raclopride in a study on living mice: both dopamine depletion by reserpine and dopamine increase by amphetamine had greater effects on the binding potential of tritiated NPA. A study in anesthetized Göttingen miniature pigs, using compartmental analyses, showed fast tracer metabolism, compensated by high brain uptake during the first minutes; the striatal binding potential was comparable with [^11^C]raclopride, and both MPTP pretreatment and deep brain stimulation of the subthalamus failed to produce obvious effects on [^11^C]NPA binding *in vivo* (Cumming et al., 2003). Another quantitative study in baboon validated the use of model-based approaches to quantify [^11^C]NPA binding *in vivo* (Hwang et al., 2004). Other PET studies in baboons confirmed its higher sensitivity to dopamine release in striatum compared to [^11^C]raclopride (Narendran et al., 2004; Hwang et al., 2005). The proportion of D_2_ receptors at high affinity was estimated to be 79% in the striatum using a bolus plus constant infusion (Narendran, 2005). A study in 1 male baboon did not show significant difference between time of recovery after amphetamine-induced dopamine release as measured by [^11^C]raclopride and [^11^C]NPA (Narendran et al., 2006). Subsequent studies focused on translation to humans. An initial study concluded that administration of a common dose of radiotracer (370 Mbq) yielded an acceptable dosimetric range in all organs (Laymon et al., 2009). Reproducibility studies and kinetic modeling in a second human study confirmed that [^11^C]NPA was a reliable tool to image D_2_/D_3_ receptors in high-affinity state in striatum (Narendran et al., 2009). Narendran and colleagues performed a comparative evaluation of [^11^C]NPA and [^11^C]raclopride to measure amphetamine-induced dopamine release in the human striatum after oral amphetamine pretreatment: decreases in BP_*ND*_ level were slightly greater for the agonist radiotracer, whereas no significant difference was found for BP_*P*_ (Narendran et al., 2010). McCormick et al. (2010), in *ex vivo* studies, demonstrated that isoflurane increased the binding and amphetamine sensitivity of [^11^C]NPA and other agonists in comparison with [^11^C]raclopride, which may have been a confounding factor in several preclinical studies that reported higher sensitivity of agonist radiotracers to endogenous dopamine.

An initial clinical study in cocaine abusers versus controls did not find any differences in D_2_/D_3_ binding in striatum, contrary to several studies that previously reported lower binding of [^11^C]raclopride in cocaine abusers. The authors concluded that D_2_/D_3_ receptors in high-affinity state may be unaltered in cocaine dependence (Narendran et al., 2011). Other preclinical studies were performed using [^11^C]NPA: one *ex vivo* study demonstrated that it was more effective than [^11^C]raclopride in detecting an increase in receptor availability following unilateral injections of 6-OH-DA in rat, a classical model of Parkinson’s disease (Palner et al., 2011); another study in rat suggested differential distribution of tritiated NPA and raclopride in the striatum, with comparable B_*max*_ in the dorsal striatum and lower B_*max*_ for [^3^H]NPA in the ventral striatum (Minuzzi and Cumming, 2010).

#### [^11^C]MNPA

MNPA, a methoxy-NPA derivative, was initially described in Gao et al. (1990), and was characterized as a D2R agonist (K_*i*_ = 0.17 nM). A preliminary PET study in cynomolgus monkey in Halldin et al. (1992) demonstrated high binding of [^11^C]MNPA in the striatum. Finnema et al. (2005) described an optimized radiosynthesis and further *in vivo* PET experiments in cynomolgus monkey. The study confirmed previous findings about D_2_ specificity: pretreatment with unlabeled raclopride considerably decreased the signal in regions known to contain D_2_ receptors. Two advantages were mentioned in comparison with [^11^C]NPA: a fivefold higher affinity, which might enable quantitative analysis in extrastriatal regions such as thalamus, and easier radiosynthesis of [^11^C]MNPA, which only needs [^11^C]methylation. Metabolite analysis did not show any lipophilic metabolites which could interfere with the parent-compound signal in the brain. The sensitivity of the radiotracer to synaptic dopamine levels was then compared versus [^11^C]raclopride in cynomolgus monkeys; [^11^C]MNPA showed higher sensitivity. Using the same methodology of high-affinity state quantification proposed by Narendran with [^11^C]NPA, the authors suggested that about 60% of D_2_ receptors were in the high-affinity state (Seneca et al., 2006). In preparation for future human studies, a kinetic brain analysis and whole-body imaging study was performed in monkeys (Seneca et al., 2008a). Brain distribution volumes were identified using a 2-tissue compartment model and in accordance with the known distribution of D_2_/D_3_ receptors, and the estimated dosimetry in human, extrapolated from the preclinical results, was moderate to low. However, the authors reported that BP_*ND*_ values of [^11^C]MNPA were lower than [^11^C]raclopride or other agonist radiotracers such as [^11^C]PHNO and [^11^C]NPA, probably due to higher uptake in the cerebellum. Seneca et al. (2008b), the same team conducted a PET study in rat using a bolus/infusion paradigm following dopamine depletion pretreatment with reserpine plus alpha-methyl-para-tyrosine. Dopamine occupancy at baseline was estimated at 53% in rat brain, in agreement with previous results with other agonist radiotracers. In the same study, binding in striatum was displaceable with raclopride but not by BP897, a selective D_3_ ligand, suggesting that [^11^C]MNPA binds specifically to D_2_ and not D_3_ receptors. Skinbjerg et al. (2009) conducted a precise pharmacological characterization of MNPA with *in vitro* studies on recombinant cells and membrane preparations. They concluded that MNPA was a full agonist of D_2_ and also D_3_ receptors (in contrast to previous *in vivo* findings). Also, two high- and low-affinity binding states were observed only for membrane preparations and not for cells. Other preclinical studies were conducted the same year: Finnema et al. (2009) determined the occupancy of the agonist apomorphine at increasing doses in cynomolgus monkey using [^11^C]raclopride and [^11^C]MNPA. Contrary to the hypothesis that agonist radioligands are more sensitive than antagonists to competition with pharmacological doses of agonists, there was no difference between Ki and ID50 determined with both radiotracers, suggesting that all D_2_/_3_ receptors are in high-affinity state or that there is only a single receptor state. Tokunaga et al. (2009) reported an example of a drug discovery approach using *in vivo* imaging with [^11^C]MNPA to detect dopamine neurotransmission system modulation by MPEP, an antagonist of group mGlu_1_ receptors. Steiger et al. (2009), an optimization of radiosynthesis was also described, with a time of about 40 min after radionuclide production.

The first clinical trial, on 10 subjects, in Otsuka et al. (2009), used a classical protocol with arterial blood sampling and metabolite analysis (and PET procedure). Results showed a distribution pattern in concordance with the D_2_ receptor distribution. The SRTM and transient equilibrium methods were validated to estimate binding potentials. Another clinical study investigated binding of the antipsychotic risperidone on high- or low-affinity D_2_ receptors with [^11^C]raclopride and [^11^C]MNPA and found that risperidone bound indifferently to both states of D_2_ receptors, with comparable occupancies and ED50 values for both tracers (Kodaka et al., 2010). In 2013, a study investigated the reproducibility of the binding potential ratio of [^11^C]MNPA to [^11^C]raclopride, reflecting the proportion of receptors in high-affinity state compared to overall receptor density; reproducibility was satisfactory in the caudate and putamen (Kodaka et al., 2012). More recently, the same team studied the different D_2_ receptor affinity states in 11 antipsychotic-free schizophrenic patients, using [^11^C]raclopride and [^11^C]MNPA; the binding potential ratio (agonist/antagonist) was significantly higher in the putamen in patients than control subjects, despite unchanged levels of total D_2_ receptors (Kubota et al., 2017).

Other preclinical studies using [^11^C]MNPA were conducted over the years. Skinbjerg et al. (2010) showed that *in vivo* striatal binding of the tracer was unchanged in dopamine beta-hydroxylase-deficient mice, concluding that their increased sensitivity to psychostimulants was not due to a higher proportion of receptors in the high-affinity state. The same year, the D_2_ receptor occupancies of quinpirole, aripiprazole, and haloperidol were estimated in conscious rats, using tritiated MNPA, PHNO, and raclopride (Peng et al., 2010). All compounds showed similar occupancy values with the different radioligands, presumably due to a high proportion of receptors in the high-affinity state *in vivo*. Another study, focusing on stress in conscious monkeys, showed that stress level correlated negatively with [^11^C]raclopride binding, and positively with [^11^C]MNPA binding (Tsukada et al., 2011).

#### [^11^C]PHNO

The first radiolabeling and preclinical evaluation of the napthoxazine derivative D2-agonist (+)-4-Propyl-3,4,4a,5,6,10b-hexahydro-2H-naphtho[1,2-b]-[1,4]-oxazin-9-ol, or (+)-PHNO, was reported in Wilson et al. (2005). [^11^C]-(+)-PHNO binding in rat brain was highly selective and specific to D2 receptors. The tracer also showed sensitivity to increases and decreases in endogenous dopamine levels. The full D_2_ agonistic properties of (+)-PHNO were previously documented (Jones et al., 1984). The first-in-man study was performed 1 year later (Willeit et al., 2006). [^11^C]-(+)-PHNO displayed good brain uptake and favorable kinetics; test–retest data suggested BP estimates to be reliable, and pre-treatment with haloperidol reduced specific binding without detectable changes in cerebellum, validating its utility as a D_2_-receptor agonist radioligand for PET. Binding in the globus pallidus was greater than with the D_2_ antagonist [^11^C]raclopride, suggesting that [^11^C]-(+)-PHNO also binds significantly to D_3_ receptors in humans. Tracer distribution, displaceability by endogenous dopamine, specificity and modeling properties were further explored in cat and compared versus [^11^C]raclopride (Ginovart et al., 2006a). Although Scatchard analysis showed comparable Bmax values with both [^11^C]-(+)-PHNO and [^11^C]-raclopride, the agonist was more sensitive than the antagonist to dopamine release (BP inhibition up to 83 versus 56%, respectively). The signal-to-noise ratio in the striatum was also 2.5-fold higher than that of [^11^C]NPA. Unusually high binding in the globus pallidus was reported in baboons and explained by higher affinity of [^11^C]-(+)-PHNO for D_3_ receptors than other D_2_/D_3_ radioligands, possibly contributing to its greater vulnerability to endogenous dopamine (dopamine affinity being higher for D_3_ than D_2_ receptors) compared to other radioligands (Narendran et al., 2006).

Kinetic modeling of the tracer was then described in humans (Ginovart et al., 2006b), to enable [^11^C]-(+)-PHNO binding to be quantified in clinical studies. Willeit et al. (2008), the D_2_–D_3_ agonist radioligand was reported to be sensitive to competition with endogenous dopamine in humans. Several studies were then performed with [^11^C]-(+)-PHNO, to study the high-affinity state of D_2_ receptors, D_3_ receptors and endogenous dopamine release in schizophrenia, addiction or according to social status (Graff-Guerrero et al., 2008; Mizrahi et al., 2011, 2012; Le Foll et al., 2013; Matuskey et al., 2015; Caravaggio et al., 2016). This radiotracer was often compared to [^11^C]raclopride, but it is unclear if the differences between the two radiotracers are due to the agonistic properties of [^11^C]-(+)-PHNO or to its higher affinity for D_3_ receptors. For instance, [^11^C]-(+)-PHNO shows preferential uptake in the ventral striatum and globus pallidus, due to the high density of D_3_ receptors in these areas, whereas [^11^C]raclopride shows preferential uptake in the dorsal striatum; [^11^C]-(+)-PHNO wash-out is also slower in the globus pallidus compared to the other regions (Graff-Guerrero et al., 2008). Similarly, a PET study in Parkinson’s disease showed a significant decrease in [^11^C]-(+)-PHNO levels in the globus pallidus, in contrast to [^11^C]raclopride, and an agonist/antagonist ratio that decreased proportionally to motor deficit and lowered mood, interpreted as the consequence of D_3_ receptor density modifications (Boileau et al., 2009). Searle et al. (2010) suggested that [^11^C]-(+)-PHNO uptake is due to high-affinity D_2_ receptors in the dorsal striatum, to high-affinity D_2_ receptors and D_3_ receptors in the ventral striatum, globus pallidus and thalamus, and only to D_3_ receptors in the substantia nigra). Numerous preclinical studies were also performed using [^11^C]-(+)-PHNO. Seeman et al. (2007) reported a 2-to-3-fold increase in high-affinity D_2_ receptors in rat following repeated injection of amphetamine, explaining why the animals were more sensitive to dopaminergic agonists. One year later, the sensitivity was compared between [^11^C]-(+)-PHNO and [^3^H]raclopride after various pharmacological challenges in conscious rats (McCormick et al., 2008). Similar degrees of inhibition were shown for both radiotracers following the pre-injection of amphetamine, cold NPA (a full agonist), aripiprazole (a partial agonist), haloperidol and clozapine (D_2_ antagonists). However, these results were contradicted by another study that showed greater *ex vivo* inhibition of [^3^H]PHNO binding by NPA than by [^3^H]raclopride, and greater displacement of the agonist radiotracer in amphetamine-sensitized rats (Kumar et al., 2006; Seeman, 2009). Contrasting results were published the same year by McCormick et al. (2009), underlining significant discrepancies in the field which require further study.

### D_4_ Receptors

Although no convincing radiotracer specific to D_4_ receptors is presently available for PET neuroimaging in the human brain, the discovery of an inverse agonist was reported in Prante et al. (2008). The compound was derived from FAUC 113 and FAUC 213 and was more selective for D_4_ receptors than for D_2_ and D_3_ receptors. The [^18^F]-labeled molecule showed specific binding in rat brain *in vitro*, but no further investigations have yet been reported.

### Serotonergic Receptors

Serotonin neurotransmission is characterized by a large number of subfamilies of receptors (14 are currently described). After the dopaminergic system, it is the second neurotransmission system to have benefited from the development of many PET radiotracers. While a significant number of them can be used in humans as radiopharmaceuticals, most are antagonists and currently very few agonists are available. However, the therapeutic potential of these many sub-families of receptors in psychiatry and neurology justifies further research in this area.

### 5-HT_1A_ Receptors

#### From [^18^F]F15599 to [^18^F]F13640

Much effort has been made to develop a radiotracer agonist of 5-HT_1A_ receptors, with initially limited success. These attempts included exploration of derivatives of various structures (analogs of 8-OH-DPAT and apomorphine, arylpiperazine, or thiochromine based ligands) which showed promising properties *in vitro* but were generally not suitable for *in vivo* imaging because of lack of evidence of specific binding (Thorell, 1995; Mathis et al., 1997; Suehiro et al., 1998; Hwang et al., 2001; Fujio et al., 2002; Zimmer et al., 2003; Vandecapelle et al., 2004). Lemoine et al. (2010), the well-characterized agonist F15599, initially seen as a drug candidate, was radiolabeled with [^18^F] and explored by PET imaging in rats and cats. It showed high affinity (K_*i*_ = 2.2 nM) and excellent specificity for 5-HT_1A_ receptors, acting as a full agonist both *in vitro* and in pharmacological tests in rats, with preferential activity at post-synaptic receptors (Newman-Tancredi, 2011). Despite encouraging *in vitro* results, the signal-to-noise ratio was insufficient for PET imaging. Its structural analog, [^18^F]F13714, displaying higher affinity for 5-HT_1A_ receptors (K_*i*_ = 0.1 nM), was also evaluated (Lemoine et al., 2012). Despite a better SNR and evidence of binding to 5-HT_1A_ receptors *in vivo*, [^18^F]F13714 binding was irreversible in rat, cat and rhesus monkey, which suggests it would be difficult to quantify binding parameters in humans. Interestingly, [^18^F]F13714 was also compared with the antagonist [^18^F]MPPF in conscious and anesthetized marmosets; it displayed a markedly different distribution pattern from [^18^F]MPPF, with highest uptake in raphe and cortical areas, as opposed to hippocampus and amygdala for the antagonist radiotracer. It also showed region-specific uptake changes in isoflurane-anesthetized animals, contrary to [^18^F]MPPF for which global increase throughout the brain was observed (Yokoyama et al., 2016). Top design a radiotracer that would be easier to quantify, the structural analog [^18^F]F13640 was recently evaluated (Vidal et al., 2018). F13640 also exhibits high selectivity for 5-HT_1A_, but intermediate affinity (K_*i*_ = 1 nM) compared to the previous two attempts. [^18^F]F13640 showed specific binding to 5-HT_1A_ receptors and agonistic properties *in vitro* and *in vivo* in rats, cats and rhesus monkeys, despite a distribution pattern contrasting with antagonist radiotracers (and similar to that of [^18^F]F13714). Moreover, *ex vivo* autoradiography experiments using pharmacological challenge with d-fenfluramine in rats suggested that [^18^F]F13640 is far more sensitive to competition with endogenous serotonin than the antagonist [^18^F]MPPF. Despite a slow washout, tracer binding is reversible, with increased washout after administration of fenfluramine, a serotonin releaser, during scanning in rats and cats (unpublished data). An autoradiographic study performed on postmortem hippocampus slices from AD patients at different Braak stages also demonstrated a decrease in [^18^F]F13640 binding in the CA1 area, occurring earlier than the decrease in [^18^F]MPPF binding in AD subjects. Further studies are ongoing to characterize the properties of [^18^F]F13640, and a first-in-man study is currently underway (clinicaltrials.gov number: NCT03347331). The first images in human showed a binding pattern different from that seen with the conventional antagonist 5-HT_1A_ radiopharmaceutical [^18^F]-MPPF (Colom et al., 2019).

### 5-HT_2A_ Receptors

#### From [^11^C]Cimbi5 to [^11^C]Cimbi36

Ettrup et al. (2010) reported [^11^C]-labeling and evaluation of the *N*-benzyl phenylethylamine derivative Cimbi-5, previously described as a selective and very potent agonist for 5-HT_2A_ receptors (Braden et al., 2006). [^11^C]Cimbi-5 distribution was consistent with the known 5-HT_2A_ distribution, and it was blocked by the antagonist ketanserin in pig brain (Ettrup et al., 2010). In an attempt to optimize the target-to-background binding ratio, the same team evaluated 9 other phenylethylamine analogs of [^11^C]Cimbi-5 in pig (Ettrup et al., 2011). The analog [^11^C]Cimbi-36 was identified as the most promising candidate for PET imaging of 5-HT_2A_ receptors as it showed the highest target-to-background binding ratio and was displaceable by ketanserin. *In vitro* studies confirmed that Cimbi-36 was a potent and selective 5-HT_2A_ agonist (K_*i*_ = 1 nM, ED_50_ = 0.5 nM). Its properties were then characterized in non-human primate and compared with the antagonist [^11^C]MDL-100907 (Finnema et al., 2014). [^11^C]Cimbi-36 distribution was again consistent with the known 5-HT_2A_ distribution and blocked by ketanserin in all brain regions except the cerebellum, which was found to be a suitable reference region. Binding potential was approximately half that of [^11^C]MDL-100907 across cortical regions but higher in other brain regions such as the choroid plexus, which was found to be related to 5-HT_2C_ receptor binding as it was blocked by the 5-HT_2C_ ligand SB 242084. The authors concluded that [^11^C]Cimbi-36 was an agonist radioligand suitable for examination of 5-HT_2A_ receptors in cortical regions and of 5-HT_2C_ receptors in the choroid plexus of the primate brain. The first-in-man study was performed in 29 healthy volunteers, with arterial input measurement and pretreatment with ketanserin in 5 subjects (Ettrup et al., 2014). The authors concluded that [^11^C]Cimbi-36 was a suitable agonist radioligand for PET imaging of high-affinity 5-HT_2A_ receptors in the cortex, and that cerebellum was an appropriate reference tissue for quantification without blood sampling. Recently, test–retest reproducibility was investigated and the distribution was compared to the antagonist [^18^F]altanserin in humans (Ettrup et al., 2016). The results showed excellent test–retest reproducibility and a high correlation between the two radiotracers, Cimbi-36 and altanserin, except in regions with high 5-HT_2C_ receptor density (choroid plexus and hippocampus), where [^11^C]Cimbi-36 binding is probably to both 5-HT_2A_ and 5-HT_2C_ receptors. Sensitivity in detecting changes in endogenous 5-HT levels was also explored in pig brain using various pharmacological challenges, by simultaneous measurement of extracellular 5-HT concentration with microdialysis and PET imaging (Jorgensen et al., 2017). There was a significant correlation between 5-HT levels and 5-HT_2A_ occupancy, indicating that [^11^C]Cimbi-36 is sensitive to competition with serotonin, although only at sufficiently high release. Another study in rhesus monkey demonstrated significant decrease in [^11^C]Cimbi-36 binding in most brain regions following administration of fenfluramine at 5 mg/kg (Yang et al., 2017). The tracer was found to be more sensitive to 5-HT release than the antagonist [^11^C]MDL 100907, and with sensitivity comparable to [^11^C]AZ10419369, a 5-HT_1B_ partial agonist currently considered to be one of the most sensitive radioligands.

Finally, a study in humans recently compared two positions of [^11^C]-labeling for the tracer and concluded that the position initially chosen in the previous studies produced a higher signal-to-noise ratio (Johansen et al., 2018).

### Cannabinoid Receptors

The endocannabinoid system is more recent in discovery compared to previous monoaminergic systems. This neurotransmission system seems to play key modulatory roles in the brain and much effort has been made to try to understand precisely its pathophysiological role in various behavioral and neurological diseases. While there are currently two known subtypes of cannabinoid receptors, termed CB_1_ and CB_2_, only the first have benefited from the development of agonist radiotracers.

### CB_1_ Receptors

#### [^11^C]JHU75528 or [^11^C]OMAR

Fan et al. (2006) reported the synthesis of [^11^C]JHU75528, to image CB_1_ receptors. The tracer showed higher *in vitro* affinity and lower lipophilicity than the two prototypical CB_1_ agonists, rimonaban and AM281). Autoradiography studies in mice and PET studies in baboon showed high cerebral uptake, good SNR and specific binding displaced by the cold ligand or rimonaban pretreatment. Metabolite analysis demonstrated that a few fractions cross the BBB (Horti et al., 2006). The first clinical assays on humans confirmed a good CB_1_ receptor quantification (Horti, 2007; Wong et al., 2008). In terms of quantification, plasma reference graph analysis (Logan et al., 1990) was found to be more reliable than the two-compartmental model for estimating Vt. It was not possible to use pons or white matter as reference regions, due to small size and lack of favorable kinetics, respectively (Wong et al., 2010). Comparison between healthy volunteers and schizophrenic patients found elevated values in patients, especially in the pons region. This preliminary study showed the potential of Vt values to prove CB_1_ involvement in schizophrenia and to predict the type and severity of clinical symptoms. Gao et al. (2012) proposed a new synthesis route for [^11^C]OMAR and analogs, and Normandin explored more precisely the quantification of the tracer in humans (Normandin et al., 2015). They found that multilinear analysis was the most robust method. Test–retest reproducibility was satisfactory. There were significant sex differences in tracer properties, and especially in metabolism and brain uptake. These findings suggest that [^11^C]OMAR is a reliable radiotracer and that, in this case, gender differences must be considered in PET analysis.

#### [^18^F]MK-9470

Continuing previous efforts to develop a CB_1_ receptor radiotracer, Burns et al. (2007) developed [^18^F]MK-9470, a specific inverse agonist, in a context of drug development. The *in vitro* affinity of MK-9470 was 0.7 nM with a 60-fold selectivity for CB_1_ receptors in comparison with CB_2_ receptors. Autoradiography studies on rhesus brain slices showed a signal consistent with CB_1_ receptor distribution. PET studies in monkeys showed rapid uptake with displaceable binding by the specific inverse agonist MK-0364. *In vivo* PET study in humans showed slow kinetics with high uptake in striatum, frontal cortex and posterior cingulate. Metabolite analysis and test–retest reproducibility were satisfactory enough to envisage [^18^F]MK-9470 as a suitable radiotracer to explore CB_1_ receptor density. These findings were applied to determine the occupancy of the inverse agonist MK-0364. One year later, a biodistribution and radiation dosimetry study demonstrated acceptable dose exposure and the feasibility of multiple scans (Van Laere et al., 2008b). The tracer was also used to assess gender differences in CB_1_ receptor distribution and changes in receptor expression with healthy aging (Van Laere et al., 2008a). Several clinical studies were then performed, including a drug occupancy study of the CB_1_ receptor inverse agonist taranabant (Addy et al., 2008), and studies exploring the relationship between CB_1_ receptors and personality traits (Van Laere et al., 2009), temporal lobe epilepsy (Goffin et al., 2011), Parkinson’s disease (Van Laere et al., 2012), eating disorder (Gérard et al., 2011; Ceccarini et al., 2016), migraine (Van der Schueren et al., 2012), schizophrenia (Ceccarini et al., 2013), AD (Ahmad et al., 2014), chronic cannabis use (Ceccarini et al., 2015), alcohol abuse (Ceccarini et al., 2014), and Huntington’s disease (Ceccarini et al., 2019). The tracer was also used in numerous preclinical studies (Goffin et al., 2008; Casteels et al., 2010a,b,c,d, 2011, 2014; Gérard et al., 2010; Ooms et al., 2014; Cleeren et al., 2018). Several studies were also performed to optimize quantification in humans (Sanabria-Bohórquez et al., 2010) and rats (Casteels et al., 2012; Miederer et al., 2018) and radiosynthesis (Thomae et al., 2014).

### Muscarinic Receptors

Muscarinic acetylcholine neurotransmission have been described for several decades and its interest has resurfaced more recently because of their implications in neurodegenerative diseases, justifying research work in PET neuroimaging. Five subtypes of muscarinic receptors have been determined, named M_1_–M_5__._ Among them, only the M_2_ family has benefited from the development of agonist PET radiotracers used in humans.

### M_2_ Receptors

#### [^18^F]FP-TZTP

In this context, Sauerberg et al. (1992) developed a series of muscarinic agonists containing a thiadiazolyl moiety. Based on these data, three fluorinated derivatives of TZTP were evaluated *in vitro* for their affinity toward the various muscarinic receptors (Kiesewetter et al., 1995). The derivative FP-TZTP displayed higher affinity for M2 than M1 receptors (K_*i*_ = 2.2 vs. 7.4 nM) and was radiolabeled with [^18^F] and further evaluated in rat. [^18^F]FP-TZTP displayed specific binding, being dose-dependently blocked by the analog P-TZTP, also a M2-preferring agonist, but only partially blocked by antagonists of M1 or M2 receptors., [^18^F]FP-TZTP was then evaluated in preclinical *in vitro* and *in vivo* studies in rats and monkeys: [^18^F]FP-TZTP showed specific binding to M2 receptors, significantly inhibited by cold compound and L-687,306, a muscarinic agonist. Uptake was significant in cortical and subcortical regions, with low uptake in cerebellum. Metabolism study in rats showed no significant presence of radiometabolites in the brain (Kiesewetter et al., 1999). The 1-compartmental model was the most reliable for determining distribution volume in rhesus monkey (Carson et al., 1998). Finally, [^18^F]FP-TZTP was sensitive to variations in ACh levels induced by physostigmine, an AChEI. Kiesewetter et al. (2003) reported 1-step automated radiosynthesis of [^18^F]FP-TZTP, and Ma et al. (2003) described a method using liquid–liquid PE and solid phase extraction to rapidly measure concentrations of tracer and metabolites thanks to the previous identification of the metabolite structures by LC-MS-MS (Ma et al., 2002).

The first-in-man study was performed in Podruchny et al. (2003) on healthy young and older volunteers. The binding pattern of [^18^F]FP-TZTP was consistent with the known M2 receptor distribution. Older subjects had significantly greater distribution volumes, which was explained by lower synaptic acetylcholine concentrations. Jagoda et al. (2003) used different models of KO mice to confirm the M2 selectivity of [^18^F]FP-TZTP, demonstrating a significant decrease in binding (from 51 to 61%) only in M2R KO mice, almost none in M1R KO mice (about 20% in amygdala and hippocampus), and none in M3R and M4R KO mice. Considering the fact that P-TZTP and the cold agonist FP-TZTP used in competition studies could produce changes in cerebral blood flow, decreasing the PET signal by reduced tracer delivery rather than by competition for receptors, Shimoji et al. (2003) showed that inhibition of [^18^F]FP-TZTP by these compounds was not due to agonist-induced reduction in CBF: the degree of tracer uptake inhibition was unchanged when a peripheral muscarinic antagonist was combined with muscarinic agonists to prevent the CBF changes induced by agonists alone. In a new clinical study, [^18^F]FP-TZTP was used to compare two populations of healthy subjects with and without apolipoprotein E-epsilon 4 allele, which is associated with increased susceptibility to AD and age-related memory problems (Cohen et al., 2003). APOE-epsilon4+ subjects had greater distribution volumes in gray matter than APOE-epsilon4- subjects, which was again interpreted in terms of synaptic acetylcholine concentration differences. [^18^F]FP-TZTP was then used to understand the cholinergic contribution to the emotional and sensory effects of procaine. Procaine dose-dependently decreased [^18^F]FP-TZTP specific binding (Benson et al., 2004). Following the 2003 clinical study, a new study was performed to evaluate the influence of age and APOE-epsilon4 genotype on the increase in acetylcholine concentration induced by physostigmine infusion and the distribution volumes of [^18^F]FP-TZTP (Cohen et al., 2006). It was also demonstrated that physostigmine induced a decrease in [^18^F]FP-TZTP uptake, and that both age and APOE-e4 genotype influenced the modulation of PET signal by physostigmine infusion. Furthermore, [^18^F]FP-TZTP was used to demonstrate the involvement of M2 receptors in mood disorders: there was decreased binding in patients suffering from bipolar disorder, which could be due to a reduction in M2 receptor density or affinity, or to an increase in endogenous acetylcholine levels (Cannon et al., 2006). van Oosten et al. (2009) reported an optimized radiosynthesis using a new precursor. Cannon et al. (2011), another clinical study was performed combining [^18^F]FP-TZTP-PET and genetic analyses: it was shown that single nucleotide polymorphisms for the M2R gene were associated with changes in [^18^F]FP-TZTP binding in bipolar disorder patients. Finally, Ravasi et al. (2012) found that constant infusion of [^18^F]FP-TZTP was better than bolus injection for performing microPET in rodents: blood clearance and metabolism were too rapid to measure a reproducible input function after bolus injection.

### Histaminergic Receptors

There are four known histamine receptors, H_1_, H_2_, H_3__,_ and H_4_. The first imaging works focused on the H_1_ receptor but without the development of PET agonists. More recently, H_3_ receptor, a target with emerging pathophysiological implications, has led to the development of agonist radiotracers.

### H_3_ Receptors

The H_3_ receptor has a presynaptic location and is involved in the regulation of histamine neurotransmission and modulation of release of other neurotransmitters (Van Laere et al., 2013). Thus, it has been demonstrated that, instead of only interfering with the negative feedback loop of histamine like an antagonist, H_3_ inverse agonists potentialize histaminergic neurotransmission by decreasing constitutive H_3_ signaling. These pharmacological properties suggest new treatments for various psychiatric or neurodegenerative diseases. According to the two-state model of agonist action, inverse agonists may have higher affinity for the inactive state of the receptor (Leff et al., 1985; Berg and Clarke, 2018). Concomitant development of H_3_ receptor inverse agonist radiotracers therefore seems important for the development of new H_3_ inverse agonists as therapeutic agents, as such radiotracers may better reflect the population of receptors actually targeted by these new ligands.

#### Spiro-Isobenzofuranone Derivative: [^11^C]MK-8278

In this context, in Hamill et al. (2009) reported the radiosynthesis and evaluation of two promising inverse agonists, as shown by the inhibition of basal [^35^S]GTPgammaS binding to membrane homogenates expressing recombinant H_3_ receptor derived from a family of spiro-isobenzofuranone-based compounds (Jitsuoka et al., 2008). The study described a radiosynthesis with high specific activity and revealing appropriate *in vitro* autoradiographic distribution in rhesus monkey and human brain, and specific binding in PET experiments in rhesus monkey for both compounds, with greater brain uptake for [^11^C]MK-8278. Using a bolus plus infusion method and *in vivo* PET imaging with [^11^C]MK-8278 in rhesus monkeys, the authors also determined the occupancy of diverse H_3_ receptor inverse agonists in relation to their plasma concentration. Van Laere et al. (2014) confirmed the utility of [^11^C]MK-8278 as a specific radioligand to evaluate *in vivo* occupancy of new H_3_ inverse agonists in human brain.

They first described whole-body biodistribution and dosimetry in humans, and found that the effective dose was in the typical range of other [^11^C]-labeled radiopharmaceuticals. The binding parameters of [^11^C]MK-8278 were quantified using a metabolite-corrected arterial input function. 1TCM and SRTM methods, considering pons as a reference region, showed reproducible estimates of Vt and BP_*nd*_ values, respectively. Finally, the authors determined the human pharmacological profile of two inverse agonists, MK-024 and MK-3134, taken orally at various doses 6 h prior the PET scan; they thus obtained the receptor occupancy of both compounds as a function of oral dose or plasma concentration, demonstrating the key role of [^11^C]MK-8278 for characterizing target engagement of H_3_ inverse agonists (by calculating RO as a function of plasma concentration).

### Opioid Receptors

There are four major subtypes of opioid receptors named delta (δ), kappa (κ), mu (μ), and nociceptin receptors. Agonist radiotracers of opioid receptors development was mainly derived by radiolabeling of existing drugs and displayed extensive use to understand physiopathological mechanisms in various diseases.

### μ Opioid Receptors

#### [^11^C]Carfentanil

Radiosynthesis of the very potent μ-opioid agonist [^11^C]carfentanil was reported in Dannals et al. (1985), quickly followed by a first PET study in humans and baboons (Frost et al., 1985). High radioactivity levels were found in the striatum and thalamus and low levels in the cerebellum and occipital cortex, consistent with the known regional density of μ receptors. [^11^C]carfentanil binding was also strongly reduced by pretreatment with the antagonist naloxone, confirming its high specificity and suitability as an opioid receptor agonist radiotracer. It was then used in a clinical PET study to demonstrate elevated μ receptor concentration in temporal lobe epilepsy (Frost et al., 1988). A multicompartmental analysis was performed to quantify the binding parameters of [^11^C]carfentanil in human brain (Frost et al., 1989), and an *in vitro* binding study with the tritiated molecule further demonstrated its selectivity for the μ receptor subtype in human and rat brain (Titeler et al., 1989).

Frost et al. (1990), the binding patterns of [^11^C]carfentanil and the antagonist [^11^C]diprenorphine were compared in humans, showing different regional distributions that were explained by the non-selectivity of diprenorphine for the different subtypes of opioid receptors. In addition, a study focusing on temporal epilepsy demonstrated significant changes in opioid receptors with [^11^C]carfentanil but not [^11^C]diprenorphine (Mayberg et al., 1991). Zubieta et al. (1996), a study demonstrated the involvement of the opioid system in addiction by showing that [^11^C]carfentanil binding was increased in cocaine-dependent subjects compared to healthy controls, and correlated positively with cocaine craving. Since then, a huge number of clinical PET studies of μ receptors have been performed with [^11^C]carfentanil, focusing on epilepsy (Madar et al., 1997), the menstrual cycle (Smith et al., 1998), gender and age differences (Zubieta et al., 1999), addiction (Zubieta et al., 2000; Bencherif et al., 2004; Gorelick et al., 2005, 2008; Heinz et al., 2005; Greenwald et al., 2007; Scott et al., 2007a; Weerts et al., 2008, 2011, 2014; Ghitza et al., 2010; Ray et al., 2011; Falcone et al., 2012; Minkowski et al., 2012; Mitchell et al., 2012; Wand et al., 2012; Kuwabara et al., 2014; Domino et al., 2015; Mick et al., 2016; Nuechterlein et al., 2016; Hermann et al., 2017; Majuri et al., 2017), eating disorders (Bencherif et al., 2005; Karlsson et al., 2015, 2016; Tuominen et al., 2015; Joutsa et al., 2018), PTSD (Liberzon et al., 2007), major depression (Kennedy et al., 2006; Prossin et al., 2011; Hsu et al., 2015; Peciña et al., 2015a; Light et al., 2017) pain (Bencherif et al., 2002; Scott et al., 2007b, 2008; Wager et al., 2007; Harris et al., 2009; DosSantos et al., 2012; Hagelberg et al., 2012; Campbell et al., 2013; Martikainen et al., 2013; DaSilva et al., 2014a, b; Peciña et al., 2015b; Karjalainen et al., 2017) and behavior or emotions (Hsu et al., 2013; Mitchell et al., 2013; Nummenmaa et al., 2015, 2018; Karjalainen et al., 2016; Manninen et al., 2017; Tuulari et al., 2017; Saanijoki et al., 2018). [^11^C]carfentanil was used to measure the receptor occupancy of buprenorphin in heroin-dependent subjects (Zubieta et al., 2000; Greenwald et al., 2007), and of nalmefene in healthy subjects (Ingman et al., 2005). A multimodal study also evaluated μ receptor occupancy by the opioid receptor antagonist naltrexone and the inverse agonist GSK1521498 in relation with the modulation of the fMRI response to a food stimulus (Rabiner et al., 2011). [^11^C]carfentanil appeared to be sensitive to endogenous opioid fluctuations in studies showing decreased binding potential during somatic pain (Bencherif et al., 2002; Scott et al., 2007b), after placebo administration (Zubieta et al., 2005; Scott et al., 2008) and after pharmacological challenge associated with release of opioid peptides (Colasanti et al., 2012).

[^18^F]-labeled derivatives of carfentanil, [^18^F]fluoro-pentyl carfentanil and the analog sufentanil, [^18^F]fluoro-propyl-sufentanil were developed by Henriksen et al. (2005a). Both compounds had nanomolar affinity for μ-opioid human receptors, and their distribution in rat brain slices was consistent with μ-opioid receptor expression. The derivative of sufentanil produced almost no radioactive metabolites in mouse brain (Henriksen et al., 2005b). However, no further results have yet been reported.

### κ Opioid Receptors

#### [^11^C]GR89696 and [^11^C]GR103545

[^11^C]GR89696, a racemate that is an antagonist of κ_1_ receptors and agonist of κ_2_ receptors, was synthesized and evaluated in mice in Ravert et al. (1999). Uptake was in good agreement with known kappa opioid receptor distribution and was inhibited by kappa opioid-selective drugs. The R and S enantiomers of [^11^C]GR89696 were later characterized separately, showing that only the R enantiomer [^11^C]GR103545 exhibited selective and saturable binding to kappa receptors (Ravert et al., 2002). [^11^C]GR103545 regional binding patterns in baboon brain were also consistent with the established distribution of kappa receptors, and binding was blocked by naloxone pretreatment (Talbot et al., 2005). Another study showed that [^11^C]GR103545 also had high affinity for kappa receptors in humans *in vitro* (K_*i*_ = 0.02 nM) and in awake rhesus monkeys (Schoultz et al., 2010). K_*d*_ and B_*max*_ were estimated using a Scatchard plot in a bolus/infusion protocol, in the same species (Tomasi et al., 2013).

The first-in-man study was performed in Naganawa et al. (2014) and showed the suitability of the tracer for imaging and quantifying kappa receptors in humans, although quantification of kinetic parameters can be difficult due to lack of a reference region and to slow kinetics. Recently, a pilot study of kappa opioid receptor binding in major depression was conducted, using [^11^C]GR103545 to compare distribution volumes between healthy volunteers and patients suffering from major depressive disorder; no significant differences were detected (Miller et al., 2018). The tracer was also used to investigate the effect of various ligands on the kappa opioid receptor in rodents (Placzek et al., 2015). First, the authors validated the use of [^11^C]GR103545 to measure drug occupancy at kappa receptors by showing that specific binding was blocked by pre-injection of GR89696 and the antagonists naloxone and LY2795050. Then, they showed that injections of the kappa receptor agonist salvinorin A 1 min before the PET scan induced a dose-dependent decrease in [^11^C]GR103545 binding potential. At sufficiently high dose, this decrease persisted up to 2.5h after administration, although the half-life of salvinorin A is only few minutes, suggesting an agonist-induced adaptive response by kappa receptors. The same authors demonstrated that, although the agonist [^11^C]GR103545 and the antagonist [^11^C]LY2459989 have similar distribution patterns in rat brain, they differed in sensitivity to competition with various kappa receptor ligands (Placzek et al., 2018): the binding potential of both tracers was reduced to a similar extent by pre-injection of the opioid receptor antagonists naloxone and naltrexone, and the selective kappa receptor antagonist LY2795050, whereas other kappa antagonists blocked [^11^C]GR103545 binding more effectively (Bruchas et al., 2007). Finally, the kappa agonists butorphan and GR89696 showed comparable impact on the binding potentials of [^11^C]GR103545 and [^11^C]LY2459989, whereas the other agonists, salvinorin A and U-50488, significantly decreased [^11^C]GR103545 uptake and had no effect on [^11^C]LY2459989 (Placzek et al., 2018). The authors explained these findings by a likely different conformation recognized by LY2459989, as the mutation of the residue D138 dramatically decreased the affinity of LY2459989 and not GR103545 for kappa opioid receptors.

### Sigma Receptors

Initially considered as part of opioid receptors, pharmacological properties of sigma receptors identified them as a specific family of receptors. Two subfamilies of sigma receptors are currently identified, σ1 and σ2 receptors. If the role of σ_1_ receptors is not well-defined, potential therapeutic applications emerge in experimental neurology, justifying the research of PET agonist radiotracers.

### σ_1_ Receptors

#### [^11^C]SA4503

Kawamura et al. (1999) reported [^11^C]-radiolabeling and evaluation of SA6298, a selective σ_1_ receptor agonist. The compound showed high brain uptake *in vivo* in rats and 1 cat, but the signal was mostly non-specific. The same team evaluated the analog [^11^C]SA4503, which has slightly lower affinity but better specificity for σ_1_ receptors, with more encouraging results (Kawamura et al., 2000): there was high specific uptake in rat brain *in vivo*, as shown by blocking studies which decreased the signal proportionally to the σ_1_ affinity of the various ligands. Moreover, no radiolabeled metabolites were found in the brain. [^11^C]SA4503 binding in mouse and cat brain was also highly specific (Kawamura et al., 2000). Further experiments in conscious monkeys confirmed it as a promising radiotracer (Ishiwata et al., 2001). Although uptake increased continuously during control scans, tracer binding was displaced by haloperidol, which has high affinity for σ_1_ receptors. [^11^C]SA4503 was then used to investigate the time-course occupancy of σ_1_ receptors by haloperidol in mice (Ishiwata et al., 2003) and humans (Ishiwata et al., 2006), and its tritiated analog was used to measure age-related changes in σ_1_ receptor expression in rat brain *in vitro* (Kawamura et al., 2003a). The density of σ_1_ receptors significantly increased with age, a finding that was confirmed in a PET study in monkeys (Kawamura et al., 2003b), but not in rat brain *in vivo* by Ramakrishnan et al. (2015), who showed a decrease in BP in several brain regions in aged rats.

Mishina et al. (2005), a clinical study compared [^11^C]SA4503 binding in healthy volunteers and Parkinson’s disease patients, and found no difference between controls and patients but a significant reduction in BP in the more injured side of the anterior putamen in patients, as assessed by [^11^C]CFT binding. Quantitative analysis of σ_1_ receptors in the human brain using [^11^C]SA4503 was reported in Sakata et al. (2007). Another study was performed in AD patients: compared to elderly volunteers, AD patients had lower BP in the cortex and cerebellum (Mishina et al., 2008). The high occupancy of σ_1_ receptors by the SSRI fluvoxamine and not by paroxetine (Ishikawa et al., 2007) and high occupancy by the AChEI donepezil (Ishikawa et al., 2009) were demonstrated in living human brain at therapeutic doses. Several fluorinated analogs of [^11^C]SA4503 were synthesized and evaluated, including [^18^F]FE-SA4503 (Elsinga et al., 2002, 2004), which is non-selective for the different subtypes of sigma receptor, [^18^F]FE-SA5845, less favorable in terms of kinetics, and [^18^F]FM-SA4503, which showed high specific binding and is more selective of σ_1_ receptors (Kawamura et al., 2007).

## What Is Different With Antagonist Radiotracers?

Here, we propose to discuss the *in vivo* differences between agonist and antagonist radiotracers. The initial concept supporting the use of agonists as radiotracers is based on their preference for the high-affinity state of GPCR receptors as opposed to the total population of receptors, as observed *in vitro*. This concept should be associated to obvious differences between agonists and antagonists, such as differential sensitivity to competition with various ligands, to pharmacological alterations of G-protein/receptor coupling and to pathological alterations. Although the issue is likely to be much more complex *in vivo*, and it has proved difficult to demonstrate the existence of different coupling states of GPCR receptors in living organisms, a number of studies did highlight the above-mentioned differences. In addition, some data even suggest distinct brain distribution patterns between agonist and antagonist radiotracers for certain GPCR.

### *In vivo* Binding of Agonists Versus Antagonists

According to *in vitro* data, agonist radiotracers are expected to display lower specific binding and available receptor density than reference antagonist radiotracers. Some studies directly compared the BP (B_*max*_/K_*d*_) of an agonist and an antagonist radiotracer specific for the same target in the same subjects. For instance, Kodaka et al. (2012) compared the binding potentials of the D_2_/D_3_ receptor radiotracers [^11^C]MNPA and [^11^C]raclopride in two humans, showing that the agonist’s binding potential was about four times lower than the antagonist’s. The BP ratio between the two radiotracers was highly reproducible on test–retest, and was suggested as a possible estimate of the proportion of receptors in high-affinity state as compared to overall D_2_/D_3_R density. A similar approach was used for 5-HT_1A_ receptors, using the partial agonist [^11^C]CUMI-101 and the antagonist [^11^C]WAY-100635 in non-human primates (Kumar et al., 2012). The authors reported an average 45% lower binding potential for [^11^C]CUMI-101, with some regional variations (highest proportion of coupled receptors in the parahippocampal gyrus, and lowest in the amygdala and putamen). Another study in marmosets, comparing the full 5-HT_1A_ agonist [^18^F]F13714 and the antagonist [^18^F]MPPF, showed that antagonist binding potential was approximatively threefold higher in 5-HT_1A_R-rich regions (such as hippocampus and amygdala), whereas in striatum and thalamus BP_*ND*_ levels were similar between the two tracers (Yokoyama et al., 2016). These regional variations in the proportion of 5-HT_1A_R in high-affinity state were even greater in conscious animals. Taken together, these studies advocate differential targeting of GPCR receptors by agonists, which display lower binding potential likely because they bind preferentially to high-affinity receptor states. Therefore, if both tracers are available for a given GPCR, the proportion of highly effective receptors can be determined as compared to overall receptor density, in physiological or pathological conditions. However, interpretation of the above results is limited by a number of factors.

Firstly, comparison of binding potentials reflects the differences in B_*max*_/K_*d*_ ratio rather than B_*max*_ directly; although the affinity of radiotracers is classically known from *in vitro* binding studies, the actual *in vivo* affinity can differ significantly. Unfortunately, very few studies directly compared the *in vivo* density of receptors targeted by an agonist versus an antagonist radiotracer. Using Scatchard analyses of PET data in 2 cats, Ginovart et al. (2006a) estimated the B_*max*_ of the agonist [^11^C]PHNO to be similar to that of [^11^C]raclopride, casting doubt on differential binding of agonist/antagonist radiotracers *in vivo*. On the other hand, the B_*max*_ of [^11^C]NPA was shown to be about 79% of that of [^11^C]raclopride in baboon (Narendran, 2005).

Another problem in comparing agonists and antagonists is the selectivity of the compounds: it is rather common for them not to be fully selective for a given GPCR, complicating the interpretation of results. This is precisely the case concerning [^11^C]PHNO, which has higher affinity for the D_3_R receptor subtype than other dopaminergic radiotracers (Narendran et al., 2006). Consequently, most clinical findings using this radiotracer were interpreted in terms of D_3_R alterations rather than D2/D3R coupling state. Another example is the 5-HT_2A_R agonist radiotracer [^11^C]Cimbi-36, which displayed lower binding than the antagonist [^11^C]MDL-100907 in cortical regions in rhesus monkey, but not in the hippocampus or choroid plexus, due to significant binding to 5-HT_2C_ receptors (Finnema et al., 2014). In human brain, [^11^C]Cimbi-36 provided BPs that were comparable to (in cortical regions) or higher than (in 5-HT_2C_R-rich regions) the antagonist [^18^F]altanserin (Ettrup et al., 2016). Estimated B_*avail*_ values (knowing the plasma protein binding of each tracer and the *in vitro* affinities of each ligand) were also similar. Finally, the partial μ opioid receptor agonist [^11^C]carfentanil and the antagonist [^11^C]diprenorphine were also compared in baboons (Shiue et al., 1991) and humans (Frost et al., 1990): the greater uptake of [^11^C]diprenorphine in the striatum or cingulate cortex was explained by its significant affinity for other opioid receptor subtypes or different kinetic properties compared to [^11^C]carfentanil. In this regard, it is obvious that direct comparison of agonist and antagonist radiotracers can also be hindered by large differences in the kinetic parameters K_1_, k_2_, k_3_ and k_4_, especially as different modeling approaches may be needed to quantify BP.

Considering the existence of two affinity sites for the agonist, the kinetics of displacement by endogenous neurotransmitters or exogenous drugs differs between agonist and antagonist radiotracers. This is the case for [^11^C]raclopride and [^11^C]NPA, where quantitative autoradiography showed biphasic displacement for the agonist and monophasic displacement for the antagonist with increasing concentration of LSD (Minuzzi and Cumming, 2010). This phenomenon introduces another degree of complexity in comparing antagonist and agonist displacement in pharmacological challenge. Furthermore, it was demonstrated that activated 5-HT_1A_ receptors induced a specific dynamics on the cell surface *in vivo*, which can modify *in vivo* receptor distribution (Pucadyil and Chattopadhyay, 2007). This could explain the difference between agonist and antagonist radiotracers and also the frequent discrepancies between *in vitro* and *in vivo* data.

As the occupancy of a GPCR by its specific endogenous neurotransmitter is expected to be greater in the high- than in the low-affinity state, the estimated B_*avail*_ value for an agonist may be closer to the B_*avail*_ value for an antagonist than the theoretical B_*max*_ values, which adds another level of complexity in comparing agonists versus antagonists. Therefore, considering the number of parameters that influence radiotracer binding quantification *in vivo*, it seems reasonable to conclude that it will generally be difficult to calculate directly the ratio of coupled receptors to total receptors density reliably enough to provide meaningful pathophysiological information. Likewise, simply comparing binding potentials or even B_*avail*_ between agonist and antagonist radiotracers in physiological conditions is unlikely to answer the question of the actual existence of a high-affinity GPCR state *in vivo*.

### Greater Sensitivity to Neurotransmitter Release?

The dopamine system is the system most widely explored in terms of neurotransmitter release monitoring using PET (Finnema et al., 2015). Several PET radioligands of D_2/_D_3_ receptors are sensitive to dopamine release, such as the benzamide derivative [^11^C]raclopride, an antagonist that has been extensively used to evaluate changes in dopamine release in the striatum, providing new insights into the role of dopamine in pathological and physiological conditions. Other antagonists with higher D_2_ affinity, such as [^11^C]FLB-457 and [^18^F]fallypride, have been used to monitor extracellular dopamine fluctuations in extrastriatal regions where the density of D_2_ receptors is lower. In theory, the sensitivity of these radioligands is limited by the fact that endogenous dopamine preferentially targets the high-affinity state of D_2_/D_3_ receptors, which is only a part of total receptor density as measured by antagonist radiotracers (Laruelle, 2000). It was demonstrated that agonist radiotracers of D_2_/D_3_ receptors such as [^11^C]MNPA, [^11^C]NPA and [^11^C]PHNO were even more sensitive to DA release, both in animals (Ginovart et al., 2006a; Gallezot et al., 2014 for [^11^C]PHNO; Narendran et al., 2004 for [^11^C]NPA; Seneca et al., 2006; Skinbjerg et al., 2010 for [^11^C]MNPA and humans Narendran et al., 2010; Shotbolt et al., 2012; Caravaggio et al., 2014). These experiments determined the proportion of high- and low-affinity states of D2/D3 receptors by means of amphetamine challenge or Scatchard analysis (Table 1).

Of the numerous attempts to develop a radiotracer sensitive to serotonin release, only a few experiments with [^11^C]CUMI-101 (Milak et al., 2011) ([^18^F]F13640 (Vidal et al., 2018) and [^11^C]Cimbi36 (Jorgensen et al., 2017; Yang et al., 2017) showed sensitivity. These difficulties suggest notable differences between dopamine and serotonin competition systems: degree of receptor availability, proportion of high-affinity state receptors, and size of the accessible receptor pool (Paterson et al., 2010). However, agonist radiotracers seem to be more appropriate than antagonist radiotracers to evaluate neurotransmitter release. For example, the literature does not report significant sensitivity for the 5-HT_1A_ receptor antagonist [^18^F]MPPF, but only then in the case of a huge release of serotonin (Zimmer et al., 2002; Rbah et al., 2003), suggesting a small proportion of coupled receptors in basal state (Udo de Haes et al., 2006). Higher sensitivity to neurotransmitter release than for antagonist radiotracers was also suggested *in vivo* for [^11^C]GR103545 (for complete details, see Table 1). However, in pharmacological challenge paradigms, many agonist radiotracers lack direct comparison with antagonist radiotracers.

Finally, the effect of anesthesia has to be taken into account, particularly in preclinical studies. As observed by several teams, anesthesia is also responsible for changes in cerebral blood flow, receptor affinity and, finally, neurotransmission (Tsukada et al., 2002; Hassoun et al., 2003; Yokoyama et al., 2016). More precisely, it is also known to affect GPCR coupling (Seeman and Kapur, 2003). *In vivo* experiments on conscious subjects are consequently recommended, but assessment of such a protocol is not always possible in animals. Quick translation to human experiments is therefore desirable. Further investigation must be envisaged to explore the *in vivo* behavior of agonist and antagonist radioligands. This will certainly affect the current ligand-receptor paradigm.

### The Concept of Internalization

Internalization is a phenomenon that is induced by agonist stimulation. Briefly, variations in the neurotransmitter, especially increasing levels in the synapse, can influence receptor crossing from cell surface to intracellular compartment. This has been demonstrated for dopamine, serotonin (Riad et al., 2001), muscarinic (Keith et al., 1998) mu opioid receptors (Quelch et al., 2014), and α_2_ receptors (Olli-Lähdesmäki et al., 1999). This adaptive process can interfere with the binding of agonist radiotracers, especially in pharmacological challenge, which induces a massive release of neurotransmitter into the synapse. Thus, the observed decrease in binding following pharmacological challenge could be due to internalization more than to direct competition (Zimmer et al., 2004; Ginovart, 2005). Consequently, the level of lipophilicity could explain differences in ligand binding: lipophilic radioligands bind both free and sequestered receptors, whereas hydrophilic ligands bind receptors only at the cell surface (Aznavour et al., 2006).

For example, it has be proven that, after amphetamine challenge, the acute effect of neurotransmitter release is responsible for a large decrease in the levels of both types of radiotracer (Narendran et al., 2004; Ginovart et al., 2006a; Seneca et al., 2006). In the case of [^11^C]MNPA and [^18^F]fallypride, an antagonist, amplitude was greater for the agonist. On a short time-scale, the phenomenon of displacement was dominant; then, on a longer scale, internalization caused incomplete recovery of both radiotracers (Skinbjerg et al., 2010). However, [^18^F]fallypride is known to bind internalized receptors with affinity twofold lower than free receptors. Consequently, [^18^F]fallypride was also affected by internalization. The process of internalization remains unclear: Narendran et al. (2004) found no difference between NPA and raclopride in recovery time after amphetamine challenge.

In the case of 5-HT_2A_ receptors, Ettrup et al. (2010) found no differences in binding between the agonist [^11^C]Cimbi36 and the antagonist [^18^F]altanserin. They found a correlation between BP_*nd*_ for both radiotracers. B_*avail*_ was almost the same (164 for the agonist and 173 for the antagonist). But these results were not in agreement with *in vitro* data suggesting internalization of 80% of receptors.

### The Case of Biased Agonism

G protein-coupled receptors display two different states, an active state (coupled receptor) and an inactive state (non-coupled receptor), and it was demonstrated *in vitro* that a given receptor may be coupled to different subtypes of G protein (Offermanns et al., 1994; Laugwitz et al., 1996) depending on its location in the brain (Jin et al., 2001). In this context, studies demonstrated that an agonist can selectively trigger a single transduction pathway among the numerous transduction pathways of GPCR (Berg and Clarke, 2006; Kenakin, 2015; Luttrell et al., 2015). Consequently, agonist ligands for a single target provide their own functional signature by selecting a specific transduction pathway. This biased agonism is related to allosteric modification of the receptor defined by multiple conformations, each depending on ligand interaction with signaling proteins (Berg and Clarke, 2006; Kenakin and Christopoulos, 2012). In this context, Yokoyama et al. (2016) compared agonist and antagonist radiotracers on 5-HT_1A_ receptors in non-human primates. The binding of the biased agonist [^18^F]F13714 was not only lower than [^18^F]MPPF but very different. Although images revealed binding all consistent with 5-HT_1A_ receptor distribution (cortical regions, amygdala, hypothalamus, and raphe nucleus), there were notable differences in intensity : e.g., lower in hippocampus and amygdala and higher in the cingulate and insular cortices for the agonist radiotracer. [^18^F]MPPF showed twofold higher binding in the hippocampus and amygdala. The authors attributed these differences to the biased agonism of F13714, interacting with specific G protein subtypes and targeting a specific brain region composed of presynaptic receptors: raphe striatum and thalamus. The notion of biased agonism was also recently explored by PET/MR imaging, and contributes to defining the existence of biased agonism on 5-HT_1A_ receptors (Vidal et al., 2018). In the kappa-opioid receptor, differences in the dynamics of receptor structure induced by the agonist [^11^C]GR103545 versus the antagonists [^11^C]LY2795050 and [^11^C]LY2459989 were used to explain the *in vivo* discrepancies observed on PET imaging (Placzek et al., 2018). Biased agonism requires exploring different transduction pathways composed of a single receptor and is a key point in the hypothetical *in vivo* differences between agonist and antagonist radiotracers. *In vitro* data clearly show the existence of two different affinity states for GPCR, but there are still difficulties in demonstrating this on PET imaging, suggesting that there may be multiple receptor conformations rather than just two affinity sites. Going further, it was also demonstrated that some 5-HT_2A_ antagonists are able to trigger arrestin pathways and induce paradoxical desensitization of GPCR (Gray and Roth, 2001).

## What Is the Role of PET Agonists in Neuroimaging?

### Improving the Measure of Endogenous Neurotransmitter Release

It is assumed from the *in vitro* data that antagonists are less sensitive to neurotransmitter release than agonists. Agonists bind only to high-affinity state receptors, whereas antagonists bind to both high- and low-affinity state receptors equally. Therefore, when initiating competition, the antagonist is not effectively involved (Paterson et al., 2010; Finnema et al., 2015). On the other hand, agonist radiotracers provide direct estimation of the target affinity of endogenous neurotransmitters (Narendran et al., 2004). Considering pharmacological findings suggesting that agonists are more sensitive to neurotransmitter release, it is of great interest to test this hypothesis *in vivo*. However, as seen before, it is difficult to demonstrate high sensitivity; experimental conditions are a determining factor. Measuring neurotransmitter release involves knowing the exact neurotransmitter level, by microdialysis. The endogenous levels are too low to be estimated at baseline with a radiotracer (Finnema et al., 2015), and it is necessary to perform pharmacological challenge to obtain huge neurotransmitter release. The development of modeling of neurotransmitter release contributes to understanding these mechanisms (Normandin et al., 2012).

### Precision Pharmacology to Evaluate Neurologic Disorders and New Therapeutics

Agonist radiotracers are useful tools to develop new agonist drug candidates. In this context, it seems to be more appropriate to choose the same type of ligand when performing drug-occupancy studies. Also, in activation studies it is possible to visualize the impact of drug or task on the active population of receptors. Therefore, there is great interest in testing this clinically. Although this has not yet been formally demonstrated, it can be proposed that pathological conditions lead to a specific decoupling of GCPRs, leading to a functional deficit of neurotransmission. PET imaging by radiopharmaceutical agonists may enable precise definition of which GPCR pathways are damaged in CNS disorders and which pathways are still functional.

Firstly, it is possible to evaluate the difference in basal binding between control and pathologic conditions. Secondly, basal binding can contribute to therapeutic optimization of a drug and of early drug development by using the drug occupancy paradigm. However, for example, a study on cynomolgus monkeys found no differences in drug occupancy of apomorphine measured with [^11^C]raclopride or [^11^C]MNPA (Finnema et al., 2009). On the other hand, an *ex vivo* study showed that the D_2_ agonist NPA was more effective in detecting an increase in receptor availability in the early stages of Parkinson’s disease. Likewise, concerning 5-HT_1A_ receptors in a postmortem study in Alzheimer patients, the radiolabeled agonist F13640 was more effective in detecting transient over-expression of 5-HT_1A_ receptors, followed by functional decoupling of these same receptors, before a decrease in their total density at a later Braak stage (Vidal et al., 2016).

In therapeutics, it could be possible to stimulate only specific pathways with biased agonists, to offset GPCR damage in brain. Thus, it appears that agonist radiotracers could be useful for developing precision pharmacology (Schaffhausen, 2017), with numerous applications in pharmacotherapeutics. In any case, it is now recommended to confirm these hypotheses with *in vivo* PET studies.

### Future Challenges and Conclusion

Agonist radiotracers provide many opportunities to decipher the ligand-GPCR paradigm in neuropharmacology. It is possible to pursue *in vitro* findings with an *in vivo* design. The concomitant use of antagonist and agonist radiotracers sheds light on complex phenomena such as reversible conversion of high- to low-affinity receptors and internalization. However, the existence of two receptor affinity states has not yet been clearly demonstrated *in vivo* (Finnema et al., 2010; Skinbjerg et al., 2012). There are discrepancies between *in vitro* and *in vivo* findings, certainly because the *in vivo* environment of the neuron is complex. Study conditions are also a determining factor. Anesthesia or changes in blood flow modify radiotracer binding. There is also great variability in the methods used to quantify high-affinity receptors *in vitro* and *in vitro* (Richfield et al., 1986; De Haes, 2005; Shalgunov, 2017). The notion of biased agonism introduces more complexity in molecular effects of agonists. A combination of PET and MRI may shed light on agonist functional activities (Vidal et al., 2018). Finally, there is a need to develop new tools to demonstrate *in vivo* coupling of GPCR. Indeed, the binding of an agonist radioligand involves intracellular molecular remodeling, which is probably not the case with the binding of a “silent” antagonist. These differences in docking justify that the brain images of agonists, and in particular their distribution patterns, are not directly comparable to those currently obtained by PET imaging of receptors using mainly antagonists. It is therefore necessary to develop new models for interpreting this molecular and functional imaging of receptors. The interpretation of the models will be based in particular on the implementation of PET imaging studies that will compare the binding patterns of an antagonistic radiotracer and an agonist radiotracer directed toward the same receptor in the same subject. Studies on animal models will also provide valuable information, in particular by pharmacologically modulating the G-protein coupling state of receptors during PET acquisitions. Finally, molecular modeling, whose bioinformatics tools are constantly evolving, will make it possible to simulate the docking specificity of agonist molecules in receptor molecular niches.

All these new tools will lead to new paradigms for neuroimaging which, in turn, will contribute to new advances in neurology and psychiatry.

## Author Contributions

LZ initiated the research topic of the manuscript, proposed its initial plan and made its revision. MC and BV wrote the manuscript.

## Conflict of Interest

The authors declare that the research was conducted in the absence of any commercial or financial relationships that could be construed as a potential conflict of interest.

## Abbreviations

μ Opioid Receptors: mu opioid receptors
mGlu_1_: metabotropic glutamate receptor 1
^11^C: carbon-11
^18^F: Fluor-18
1TCM: one-tissue compartmental model
2TCM: two-tissue compartmental model
5-HT: serotonin
5-HT_1A_: subtype 1A of serotonin receptors
5-HT_1B_: subtype 1B of serotonin receptors
5-HT_2A_: subtype 2A of serotonin receptors
5-HT_2C_: subtype 2C of serotonin receptors
5-HT_4_: subtype 4 of serotonin receptors
5-HT_7_: subtype 7 of serotonin receptors
6-OH-DA: 6 hydroxy-dopamine
A_1_ Receptor: subtype 1 of adenosine receptors
A_2A_ Receptor: subtype 2A of adenosine receptors
Ach: acetylcholine
AChEI: acetylcholinesterase inhibitors
AD: Alzheimer’s disease
APOE-e4: apoenzyme 4
B_*avail*_: available density of receptors
BBB: blood–brain barrier
B_*max*_: maximal density of receptors
BP: binding potential
BP_*ND*_: non-displaceable binding potential
BPP: plasma binding potential
CB_1_: subtype 1 of cannabinoid receptors
CNS: central nervous system
D_2_: type 2 dopaminergic receptors
D_3_: type 3 dopaminergic receptors
E_*max*_: maximal effect
GABA_*B*_: subtype B of GABA receptors
GPCRs: G-protein -coupled-receptors
GTP: guanosine triphosphate
H_3_ receptors: subtype 3 of histamine receptors
IC_50_: half-maximal inhibitory concentration
Kd/Ki: affinity of receptors
KO: knock-out
LSD: diethylamide lysergic acid
M_1_R: type 1 of muscarinic receptors
M_2_R: type 2 of muscarinic receptors
M_3_R: type 3 of muscarinic receptors
M_4_R: type 4 of muscarinic receptors
nor-BNI: norbinaltorphimine
PET/fMRI: Positron emission tomography/functional magnetic resonance imaging
PET: Positron emission tomography
PgP: P glycoprotein
SRTM: simplified tissue reference model
κ Opioid Receptors: kappa opioid receptors
σ_1_: sigma 1 receptors
