# Supplementary material for: Is There a Role for GPCR Agonist Radiotracers in PET Neuroimaging?
Source: Front Mol Neurosci. 2019 Oct 18;12:255. doi: 10.3389/fnmol.2019.00255 (PMC6813225; doi:10.3389/fnmol.2019.00255)
Supplement: Supplementary file 1 [file Data_Sheet_1.docx]

# Supplementary Material

## The following paragraphs describe the PET agonist radiotracers whose preclinical studies have not yet led to imaging studies in humans.

## Dopaminergic receptors

#### D1 receptors

##### [^11^C]SKF 82957/[^11^C]SKF 75670

In 1996, DaSilva and colleagues reported the synthesis and in vitro autoradiographic evaluation of benzazepine derivatives known for their D_1_ agonist properties, radiolabeled with ^11^C: i.e., the partial agonist [^11^C]SKF 75670 and its 6-chloro derivative agonist [^11^C]SKF82957(DaSilva et al., 1996a). Both compounds showed specific and selective in vivo binding in rat striatum, a region rich in D_1_ receptors(DaSilva et al., 1996b). Further in vivo studies on R-[^11^C]SKF82957 demonstrated acceptable radiation dosimetry, no metabolites in rat brain extracts, and a low level of metabolites in rat plasma (DaSilva et al., 1999). However, a 2010 study in rats showed that this tracer gave rise to a brain-penetrant radiometabolite; tolcapone pretreatment increased tracer performance in imaging D_1_ receptors by inhibiting the production of this interfering metabolite (Palner et al., 2010).

#### D_2_/D_3_ Receptors

##### [^11^C]SV-III-130

[^11^C]-SV-III-130 is a partial agonist of D_2_ receptors with high affinity, and a derivative of the atypical antipsychotic aripiprazole (Vangveravong et al., 2011). Preclinical studies on rhesus monkey demonstrated selective binding to D_2_ receptors, unchanged by blocking with a D_3_ selective ligand (Xu et al., 2013). In the same study, [^11^C]SV-III-30 binding was unchanged by lorazepam pretreatment to reduce endogenous dopamine, but was decreased by administration of d-amphetamine to increase synaptic dopamine levels. Challenge studies using [^11^C]-SV-III-130 and various data on [^18^F]-LS-3134, a D_3_ radiotracer, showed that D_2_ receptors could be quantified without depleting the dopaminergic synapses of endogenous neurotransmitter, whereas that is necessary in order to quantify D_3_ receptors.

##### [^11^C]5-OH-DPAT/[^11^C]-PPHT/[^11^C]-ZYY-339

An American team aimed to develop compounds to image D_2_ high-affinity (HA) sites using the tetralin structure. They designed their first potential radiotracers labelled with ^11^C, [^11^C]-5-OH-DPAT, [^11^C]PPHT and [^11^C]ZYY-339. Incubation with sulpiride showed only a little non-specific binding in cortical areas (Shi et al., 1999). [^11^C]-5-OH-DPAT was then further evaluated *in vitro* and *in vivo*. *In vitro* studies in rats confirmed specific binding on D_2_ receptor HA sites using Gpp(NH)p (Mukherjee et al., 2004; Shi et al., 1999). *In vivo* studies in rats and monkeys found a high binding in striata with quick, reversible kinetics. Binding was also blocked by haloperidol pretreatment and increased by depletion of dopamine induced by reserpine pretreatment in rats (Shi et al., 1999). The same methodology was applied to the two other agonists [^11^C]PPHT and [^11^C]ZYY-339, with similar results but lower striatum-to-cerebellum ratios and comparable thalamus-to-cerebellum ratios (Mukherjee et al., 2004).

##### [^18^F]-5-OH-FPPAT

[^18^F]-5-OH-FPPAT, a fluorinated analog of [^11^C]-5-OH-DPAT, was designed in 2004. *In vitro* binding competition studies revealed an IC_50_ of 6.95 for this tracer, close to that of its analog 5-OH-DPAT (6.53 nM) but with higher lipophilicity. *In vitro* autoradiography studies on rat brain showed evidence of specific binding to high-affinity state D_2_ receptors using Gpp(NH)p pretreatment. *In vivo* PET imaging in rats revealed significant binding in striata, with high brain uptake. Binding studies on rhesus monkey brain slices indicated specific binding in dopaminergic regions, caudate, putamen and thalamus. These findings were confirmed by *in vivo* blockade studies in rats with the agonist PPHT or the antagonist risperidone (Shi et al., 2004). Finally, *ex vivo* autoradiography on rats with Gpp(NH)p revealed a large decrease in striatal binding, indicating high-affinity state binding of [^18^F]-5-OH-FPPAT and the newer derivative [^18^F]5-OH-FHXPAT (Mukherjee et al., 2017). Further investigations are needed to decipher more precisely the binding of these compounds to dopamine receptors.

##### AMC derivatives

Structure-activity study of 2-aminomethylchroman-7-ol (AMC) identified a number of agonists with good affinity and selectivity for the high-affinity state of D_2_ receptors (Mewshaw et al., 1997). In 2014, Shalgunov and colleagues (Shalgunov et al., 2015) tried to develop new D_2_/D_3_ agonist fluorinated radiotracers using the 2-aminomethylchroman-7-ol structure (Shalgunov et al., 2015). The fluorinated compounds [^18^]FBu-AMC13 and [^18^F]FEt-AMC15 were identified as the most promising radiotracers, with nanomolar affinities of the unlabeled compounds for D_2_/D_3_ receptors and nearly full agonism in human D_2_ receptors. *In vitro* studies revealed specific binding of both radiotracers to high-affinity D_2_/D_3_ receptors in striatum (van Wieringen et al., 2014). The team further assessed these compounds in rat brain, and other AMC-13 and AMC-15 homologs with variations on alkyl chains. All showed specific binding in autoradiography *in vitro* using raclopride. Specific binding was also decreased by an excess of GTP, demonstrating that this corresponds predominantly to the high-affinity state of D_2_/D_3_ receptors. However, *in vivo* studies showed that only a small proportion of [^18^F]FEt-AMC15 crossed the BBB. [^18^F]FBu-AMC13 showed a more optimistic profile, with good BBB penetration. Its analog [^18^F]FEt-AMC13 showed the highest signal-to-noise ratio, with similar BBB penetration and metabolic profile. However, *in vivo* blockade studies of [^18^F]FEt-AMC13 with 1 mg/kg of raclopride showed lower displacement than with other ^11^C-labeled D_2_/D_3_ tracers. Following the basic structure of (R)-2-(benzylaminomethyl)chroman-7-ol, described by Mewshaw (AMC1), the ^18^F-fluorine was attached directly to the benzyl function of AMC1 to obtain [^18^F]AMC 20 so as to improve the stability of the compound in comparison with the previous alkyl derivatives (Shalgunov et al., 2015). *In vitro* studies found it to be a potent agonist of high-affinity state D_2_ receptors, with picomolar affinity. PET imaging in rats showed good BBB penetration and accumulation in striatum. *In vivo* challenge studies with raclopride also demonstrated displacement in striatum, but still in contrast with data obtained with [^11^C]-D_2_/D_3_ agonist radiotracers: i.e., percentage occupancy on raclopride injection was lower than with [^11^C]PHNO or [^11^C]MNPA). [^18^F]AMC20 showed higher nonspecific binding than [^18^F]FEt-AMC13. Finally, the low signal-to noise ratio and a suspected lack of selectivity stopped further development of these molecules.

##### [^18^F]MCL-524

Another study focused on a fluoroalkyl derivative of [^11^C]MNPA (Finnema et al., 2014), [^18^F]MCL-524, in an attempt to develop the first fluorinated agonist radiotracer of D_2_/D_3_ receptors. MCL-524 was previously shown to have nanomolar affinity for D_2_ high-affinity receptors (Sromek et al., 2011). Finnema et al. showed that [^18^F]MCL-524 provided similar or even greater contrast between striatum and cerebellum compared to [^11^C]MNPA. Blocking studies with raclopride and D-amphetamine pre-treatment produced a decrease in binding, in agreement with a previous study with [^11^C]MNPA, confirming the specificity and agonistic profile of [^18^F]MCL-524. Radiometabolite analysis showed a metabolism rate comparable to that of [^11^C]MNPA and [^11^C]NPA; the detected radiometabolites were polar and unlikely to cross the BBB. Kinetic analysis showed a slight underestimation of BP_ND_ by the MRTM model in comparison to the 1TCM model, deserving further investigation in future human studies. A whole-body distribution study revealed that [^18^F]MCL-524 radiation dosimetry was in an acceptable range for translation to human trials, and presented an interest for simultaneous PET/fMRI protocols thanks to its longer half-life.

Fluorinated analogs of apomorphine were also synthesized by Zijlstra et al., by labeling the N-alkyl chain with fluorine-18 (Zijlstra et al., 1993b). The three compounds were evaluated in rat brain, but showed disappointing results: [^18^F]2-OH-FNPA did not cross the BBB while neither [^18^F]FNEA nor [^18^F]FNPA demonstrated specific binding to D2 receptors.

##

## Serotonin receptors

#### 5-HT_1A_ Receptors

##### [^11^C]CUMI-101 and derivatives

In 2006, the arylpiperazine derivative [^11^C]MPT was evaluated in baboon and showed specific binding to 5-HT_1A_ receptors *in vivo*, but suffered from very fast metabolism and slow washout, making quantification of binding parameters difficult (Kumar et al., 2006). Its structural analog [^11^C]CUMI-101 was then tested in baboon and reported to be a promising ligand for imaging 5-HT_1A_ receptors in high-affinity state (Kumar et al., 2007). Subsequent studies further explored its modeling properties in baboons and evaluated [^11^C]CUMI-101 in humans (Milak et al., 2008, 2010). Other studies focused on tracer sensitivity for endogenous serotonin fluctuations in non-human primates (Milak et al., 2011) and in humans (Pinborg et al., 2012), and suggested that tracer uptake is sensitive only to large increases in extracellular serotonin in response to robust pharmacological challenge but under physiological conditions. Another study in humans, using citalopram, showed a slight increase in [^11^C]CUMI-101 binding in postsynaptic regions, which was interpreted as a possible decrease in 5-HT concentration in these areas (Selvaraj et al., 2012). The authors also associated [^11^C]CUMI-101 and the antagonist [^11^C]WAY-100635 in order to quantify G-protein-coupled 5-HT_1A_ receptors compared to the total receptor pool, and reported an average of 45% of 5-HT_1A_ receptors in coupled state in baboon brain (Kumar et al., 2012). Subsequently, however, two studies questioned the specificity of the tracer, highlighting region-dependent cross-reactivity with α_1_ adrenergic receptors. Its pharmacological properties were also questioned, as it behaved as an antagonist in some *in vitro* studies (Hendry et al., 2011; Shrestha et al., 2014). Therefore, [^11^C]CUMI-101 appears rather to be a partial agonist, limiting its potential in the study of high-affinity receptors and possibly explaining its relatively poor sensitivity to serotonin fluctuation. Two recent multimodal studies also used [^11^C]CUMI-101 PET imaging in combination with fMRI, to study the presynaptic serotonergic modulation of emotional processing (Selvaraj et al., 2015) and the mechanism of action of citalopram *in vivo* (Selvaraj et al., 2018). Recent attempts were made to develop a fluorinated analog of [^11^C]CUMI-101, resulting in the synthesis of [^18^F]FECUMI-101, a partial agonist of 5-HT_1A_ receptors (E_max_=77%) with high affinity (K_i_=0.1 nM), showing specific binding in non-human primates *in vivo*, although there was unexpectedly high uptake in the thalamus (Lemoine et al., 2010; Majo et al., 2013). However, a subsequent *in vitro* evaluation of the tracer in human brain also showed significant binding to α_1_ receptors (Shrestha et al., 2014, 2016). The same team recently described the radiosynthesis of another partial agonist, [^18^F]FEMPT (Collier et al., 2017), but no preclinical evaluation of this radiotracer has yet been published.

#### 5-HT_2A_ Receptors

##### Fluorinated analogs of [^11^C]Cimbi-36

In 2016, the same team reported the synthesis and evaluation of 3 fluorine-containing derivatives of Cimbi-36. Although all were potent 5-HT_2A_ agonists, none had satisfying properties for PET neuroimaging, due either to excessive radiometabolite signal or to non-specific binding (Herth et al., 2016) . Five other analogs were evaluated and did not display specific binding, although all entered the pig brain (Petersen et al., 2016). Finally, 2 new analogs were evaluated in 2017, leading to unsuccessful displacement challenges with a 5-HT_2A_ antagonist (Edgar et al., 2017). In 2015, Prabhakaran et al. reported the synthesis of another fluorinated analog, [^18^F]FECIMBI-36. Specific binding to 5-HT_2A_ and 5-HT_2C_ receptors was shown *in vitro* on postmortem human brain slices (Prabhakaran et al., 2015). [^18^F]FECIMBI-36 was evaluated in non-human primates; results showed low brain penetration (Prabhakaran et al., 2017).

#### 5-HT_2C_ Receptors

In 2013, Granda et al. reported the synthesis and evaluation of several methylated arylazepine derivatives as potential 5-HT_2C_ agonists radiolabeled by ^11^C for PET neuroimaging. The most promising agonist was administered in baboons and showed high brain penetration but no evidence of specific binding (Granda et al., 2013). [^11^C]-radiolabelling of two potent and selective 5-HT_2C_ full agonists, WAY-163909 and vabicaserin, via Pictet-Spengler cylization, was reported in 2014. Both [^11^C]WAY-163909 and [^11^C]vabicaserin showed a high brain uptake and non-specific binding in rodent and non-human primate (Neelamegam et al., 2014).

More recently, 4-(3-[^18^F]fluorophenethoxy)pyrimidine ([^18^F]-FPP) appeared to be a promising radiotracer of 5-HT_2C_ receptors (Kim et al., 2017). This compound was previously reported to exert an agonistic effect on this receptor (E_max_=88%) and to have reasonable selectivity over other serotonin receptors (Kalgutkar et al., 2009). It showed high brain uptake and specific binding to 5-HT_2C_ receptors in rat, being decreased by co-administration of lorcaserin (although at a high dose of 10 mg/kg).

#### Other Serotonergic Receptors

No agonist radiotracer is yet available for PET neuroimaging of other serotonergic GPCR, despite several attempts with 5-HT_4_ receptors (Buiter et al., 2013) and 5-HT_7_ receptors (Lacivita et al., 2014).

## Muscarinic receptors

#### M_1_ receptor

##### [^18^F]FP-TZTP

In 1993, Halldin et al. reported [^11^C]-radiolabeling of xanomeline and butylthio-TZTP, two muscarinic agonists selective for the M_1_ receptor (Halldin et al., 1992). Both compounds were further evaluated in cynomolgus monkey and human brain as PET radioligands and drug candidate (Farde et al., 1996). They showed high brain uptake but limited washout in humans, which is not optimal for quantification of a PET radiotracer. Moreover, the low cortical-to-cerebellar ratio of both compounds and the affinity of butylthio-TZTP for σ_1_ receptors led the authors to conclude that they were not promising ligands for PET neuroimaging.

##### [^11^C]-LSN3172176

Recently, another team reported the discovery and evaluation of two selective M_1_ receptor agonists derived from oxindole, LSN3172176 and LSN3262527 (Jesudason et al., 2017; Mogg et al., 2018). High BP values were found for both molecules in *ex vivo* experiments using LC-MS-MS analysis in rats and control mice, with much lower values in M_1_ KO mice. Both agonists were radiolabeled with ^11^C and showed specific binding in rhesus monkeys *in vivo*. Further kinetic modeling experiments with [^11^C]LSN3172176 were performed in rhesus monkeys and showed reliable estimates of distribution volumes using the 1-tissue compartment model (Nabulsi et al., 2019). Scopolamine pretreatment blocked the signal, with occupancy of 98.5%. Further pharmacological characterization of [^11^C]LSN3172176 in human, rat or mouse cortical membranes showed it to be a partial agonist, with an efficacy range of 43-73% (Mogg et al., 2018).

##### [^11^C]AF150(S)

In 2013, AF150(S), an M_1_ receptor agonist analog to cevimeline, was radiolabeled with ^11^C and evaluated as a PET tracer in rodent brain (Buiter et al., 2013). The results were encouraging, with higher uptake in M_1_ receptor-rich areas and specific binding demonstrated by blocking experiments. [^11^C]AF150(S) was also sensitive to changes in endogenous acetylcholine levels induced by rivastigmine.

## Cannabinoid receptors

#### CB1 receptors

##### [^11^C]MePPEP

In 2008, Yasuno et al. reported the radiosynthesis and evaluation of [^11^C]MePPEP, ((3R,5R)-5-(3-
methoxy-phenyl)-3-((R)-1-phenyl-ethylamino)-1-(4-trifluoromethyl-phenyl)-pyrrolidin-2-one), a new inverse agonist of CB_1_ receptors (Yasuno et al., 2008). *In vitro* and *in vivo* preclinical studies showed high brain uptake in monkey brain, high specific binding on blocking studies, and rapid clearance. Quantification of distribution volumes using a 2-tissue compartment model gave satisfactory results despite the lack of identification of a reference region for simplified quantification. In a further study, the authors confirmed the previous findings regarding specificity in rodent brain (although CB_1_ knock-out mice showed about 35% non-specific binding), and that it was not a PgP substrate (Terry et al., 2008). Importantly, displacement studies showed that the tracer was more potently displaced by inverse agonists than agonists. These results suggest a large receptor reserve or different binding sites for inverse agonists and agonists. The first test in healthy human volunteers was conducted in 2009 (Terry et al., 2009) Finally, the authors developed fluorinated derivatives, and first-in-man studies with [^18^F]FMPEP-d2 demonstrated better precision and accuracy than [^11^C]MePPEP (Terry et al., 2010).

##### Other cannabinoid agonists

Two potent inverse agonist radiotracers were partially investigated through preclinical *in vitro* and *in vivo* studies, with promising results for imaging CB_1_ receptors: [^11^C]CB-119 (Hamill et al., 2009) and [^11^C]SD 5024 (Donohue et al., 2008; Tsujikawa et al., 2014). A full agonist of CB_1_ and CB_2_ receptors, AZD1490, was also radiolabeled with carbon-11 and evaluated in monkey brain for a microdosing study. The relatively low brain uptake and homogeneous distribution did not support any potential application as a radiotracer (Schou et al., 2013).

## Opioid receptors

#### κ and µ receptors

##### [^11^C]PEO

In an attempt to develop an agonist radiotracer structurally closer to [^11^C]diprenorphine, synthesis and preclinical evaluation of the full-agonist orvinol [^11^C]PEO, was reported in 2009 (Marton et al., 2009). The compound showed specific and selective binding to µ receptors in rats, and slight binding to kappa receptors. A [^18^F]-labeled derivative was synthesized (Marton and Henriksen, 2012) and evaluated in rats, and showed full-agonist properties and high affinity for all subtypes of opioid receptors (Riss et al., 2013).

#### All opioïd receptors

##### [^11^C]buprenorphine

The partial µ-agonist buprenorphine (which is also an antagonist of kappa and delta receptors) was radiolabeled with ^11^C and compared with the antagonist [^11^C]diprenorphine in mouse and baboon brain (Shiue et al., 1991). Distribution patterns were similar and consistent with the known distribution of receptors, but striatum-to-cerebellum ratio was lower with [^11^C]buprenorphine than [^11^C]diprenorphine, because of slower washout from regions devoid of receptors. Another study in baboons showed tracer displacement by naloxone, confirming it as a suitable radiotracer of opioid receptors (Galynker et al., 1996), although its non-selectivity for the different opioid receptors limits its usefulness.

##

## Adenosine Receptors

#### A_1_ Receptor

The first potential agonist radiotracer for A_1_ receptor PET imaging was developed recently (Guo et al., 2018). A series of compounds were evaluated for their affinity and selectivity for A_1_ receptors compared to other adenosine receptor subtypes, and their functional efficacy in A_1_ receptors was measured. A 3,5-dicyanopyridine derivative with good selectivity for A_1_ receptors was selected for radiolabeling with ^11^C, although its efficacy was only partial (Emax=16%). The radiolabeled molecule displayed specific binding to A_1_ receptors in rat brain *in vivo*. A fluorinated analog of adenosine, [^18^F]FNECA, was previously evaluated and showed a distribution pattern consistent with A_1_ receptors *in vitro*; however, it had very low brain uptake in rabbit *in vivo* (Márián et al., 2002).

#### A_2A_ Receptor

In 2017, a new class of fluorinated A_2A_ receptor agonists was derived, based on the potent and selective agonist ATL-313 (Lowe et al., 2017). FDA-PP1 and FDPA-PP2 displayed submicromolar affinities and agonistic properties, as sodium, which stabilizes the inactive conformation, decreased their affinities. However, no further evaluation of the radiolabeled compounds has yet been reported.

## GABA Receptors

#### GABA_B_ receptors

Contrary to the other GABA receptor subtypes, GABA_B_ receptors belong to the GPCR family. Several attempts to develop specific GABA_B_ radiotracers, including agonists, have been made. ^11^C-radiolabeling of baclofen using Michael addition of nitromethane was reported in 2009 (Kato et al., 2009) but exhibited negligible brain permeability. New fluoropyridyl ether analogs of baclofen were synthesized and evaluated recently (Naik et al., 2018). The compound showing the highest affinity and greatest agonist response, (R)-4-amino-3-(4-chloro-3-((2-fluoropyridin-4-yl)methoxy)phenyl)butanoic acid, was radiolabeled with ^18^F and studied in mice. It showed moderate brain uptake in mice and about 35% specific binding, justifying further development of improved GABA_B_ agonist radiotracers in the future.

## References

Buiter, H. J., Windhorst, A. D., Huisman, M. C., De Maeyer, J. H., Schuurkes, J. A., Lammertsma, A. A., et al. (2013). Radiosynthesis and preclinical evaluation of [11C]prucalopride as a potential agonist PET ligand for the 5-HT4 receptor. *EJNMMI Research* 3, 24. doi:10.1186/2191-219X-3-24.

Collier, T. L., Liang, S. H., Mann, J. J., Vasdev, N., and Kumar, J. S. D. (2017). Microfluidic radiosynthesis of [18F]FEMPT, a high affinity PET radiotracer for imaging serotonin receptors. *Beilstein J Org Chem* 13, 2922–2927. doi:10.3762/bjoc.13.285.

DaSilva, J., Schwartz, R., Greenwald, E., Lourenco, C., Wilson, A., and and Houle, S. (1999). Dopamine D1 agonist R-[11C]SKF 82957: synthesis and in vivo characterization in rats. *Nuclear Medicine and Biology* 26, 537–542. doi:doi:10.1016/s0969-8051(99)00015-3.

DaSilva, J., Wilson, A., Nobrega, J., Jiwa, D., and and Houle, S. (1996a). Synthesis and autoradiographic localization of the dopamine D-1. *Applied Radiation and Isotopes* 47, 279–284. doi:doi:10.1016/0969-8043(95)00306-1.

DaSilva, J., Wilson, A., Valante, M., C., H., D., W., D., et al. (1996b). In vivo binding. *Life Sciences* 58, 1661–1670. doi:doi:10.1016/0024-3205(96)00141-5.

Donohue, S. R., Krushinski, J. H., Pike, V. W., Chernet, E., Phebus, L., Chesterfield, A. K., et al. (2008). Synthesis, Ex Vivo Evaluation, and Radiolabeling of Potent 1,5-Diphenylpyrrolidin-2-one Cannabinoid Subtype-1 Receptor Ligands as Candidates for In Vivo Imaging. *Journal of Medicinal Chemistry* 51, 5833–5842. doi:10.1021/jm800416m.

Edgar, F. G., Hansen, H. D., Leth-Petersen, S., Ettrup, A., Kristensen, J. L., Knudsen, G. M., et al. (2017). Synthesis, radiofluorination, and preliminary evaluation of the potential 5-HT _2A_ receptor agonists [ ^18^ F]Cimbi-92 and [ ^18^ F]Cimbi-150. *Journal of Labelled Compounds and Radiopharmaceuticals* 60, 586–591. doi:10.1002/jlcr.3557.

Farde, L., Suhara, T., Halldin, C., Nybäck, H., Nakashima, Y., Swahn, C. G., et al. (1996). PET study of the M1-agonists [11C]xanomeline and [11C]butylthio-TZTP in monkey and man. *Dementia* 7, 187–195.

Finnema, S., Stepanov, V., Nakao, R., Sromek, A., Zhang, T., Neumeyer, J., et al. (2014). 18F-Labeled Dopamine D2 and D3 Receptor Agonist Sensitive to Dopamine: A Preliminary PET Study. *Journal of Nuclear Medicine* 55, 1164–1170. doi:doi:10.2967/jnumed.113.133876.

Galynker, I., Schlyer, D. J., Dewey, S. L., Fowler, J. S., Logan, J., Galley, S. J., et al. (1996). Opioid receptor imaging and displacement studies with [6-O-[11C]methyl]buprenorphine in baboon brain. *Nuclear Medicine and Biology* 23, 325–331. doi:10.1016/0969-8051(95)02087-X.

Granda, M. L., Carlin, S. M., Moseley, C. K., Neelamegam, R., Mandeville, J. B., and Hooker, J. M. (2013). Synthesis and Evaluation of Methylated Arylazepine Compounds for PET Imaging of 5-HT _2c_ Receptors. *ACS Chemical Neuroscience* 4, 261–265. doi:10.1021/cn300223d.

Guo, M., Gao, Z.-G., Tyler, R., Stodden, T., Li, Y., Ramsey, J., et al. (2018). Preclinical Evaluation of the First Adenosine A1 Receptor Partial Agonist Radioligand for Positron Emission Tomography Imaging. *J. Med. Chem.* 61, 9966–9975. doi:10.1021/acs.jmedchem.8b01009.

Halldin, C., Swahn, C. G., Neumeyer, J., Hall, H., Gao, Y., and Karlsson, P. (1992). Preparation of two potent and selective dopamine D-2 receptor agonists: (R)-[proply-11C]-2-OH-NPA and (R)-[methyl-11C]-2-OCH3- NPA. *Journal of Labelled Compounds and Radiopharmaceuticals*, 265–266.

Hamill, T., Sato, N., Jitsuoka, M., Tokita, S., Sanabria, S., Eng, W., et al. (2009). Inverse agonist histamine H3 receptor PET tracers labelled with carbon-11 or fluorine-18. *Synapse* 63, 1122–1132. doi:doi:10.1002/syn.20689.

Hendry, N., Christie, I., Rabiner, E. A., Laruelle, M., and Watson, J. (2011). In vitro assessment of the agonist properties of the novel 5-HT1A receptor ligand, CUMI-101 (MMP), in rat brain tissue. *Nuclear Medicine and Biology* 38, 273–277. doi:10.1016/j.nucmedbio.2010.08.003.

Herth, M. M., Petersen, I. N., Hansen, H. D., Hansen, M., Ettrup, A., Jensen, A. A., et al. (2016). Synthesis and evaluation of 18F-labeled 5-HT2A receptor agonists as PET ligands. *Nuclear Medicine and Biology* 43, 455–462. doi:10.1016/j.nucmedbio.2016.02.011.

Jesudason, C., Barth, V. N., Goldsmith, P. J., Ruley, Kevin, Johnson, M., Mogg, A., et al. (2017). Discovery of two novel, selective agonist radioligands as PET imaging agents for the M1 muscarinic acetylcholine receptor. *J Nucl Med* 58, Supplement 1 546.

Kalgutkar, A. S., Bauman, J. N., McClure, K. F., Aubrecht, J., Cortina, S. R., and Paralkar, J. (2009). Biochemical basis for differences in metabolism-dependent genotoxicity by two diazinylpiperazine-based 5-HT2C receptor agonists. *Bioorganic & Medicinal Chemistry Letters* 19, 1559–1563. doi:10.1016/j.bmcl.2009.02.032.

Kato, K., Zhang, M.-R., and Suzuki, K. (2009). Synthesis of (R,S)-[4-11C]baclofen via Michael addition of nitromethane labeled with short-lived 11C. *Bioorganic & Medicinal Chemistry Letters* 19, 6222–6224. doi:10.1016/j.bmcl.2009.08.097.

Kim, J., Moon, B. S., Lee, B. C., Lee, H.-Y., Kim, H.-J., Choo, H., et al. (2017). A Potential PET Radiotracer for the 5-HT _2C_ Receptor: Synthesis and in Vivo Evaluation of 4-(3-[ ^18^ F]fluorophenethoxy)pyrimidine. *ACS Chemical Neuroscience* 8, 996–1003. doi:10.1021/acschemneuro.6b00445.

Kumar, J. S. D., Majo, V. J., Hsiung, S.-C., Millak, M. S., Liu, K.-P., Tamir, H., et al. (2006). Synthesis and in Vivo Validation of [ *O* -Methyl- ^11^ C]2-{4-[4-(7-methoxynaphthalen-1-yl)piperazin- 1-yl]butyl}-4-methyl-2 *H* -[1,2,4]triazine-3,5-dione: A Novel 5-HT _1A_ Receptor Agonist Positron Emission Tomography Ligand. *Journal of Medicinal Chemistry* 49, 125–134. doi:10.1021/jm050725j.

Kumar, J. S. D., Milak, M. S., Majo, V. J., Prabhakaran, J., Mali, P., Savenkova, L., et al. (2012). Comparison of High and Low Affinity Serotonin 1A Receptors by PET In Vivo in Nonhuman Primates. *Journal of Pharmacological Sciences* 120, 254–257. doi:10.1254/jphs.12100SC.

Kumar, J. S. D., Prabhakaran, J., Majo, V. J., Milak, M. S., Hsiung, S.-C., Tamir, H., et al. (2007). Synthesis and in vivo evaluation of a novel 5-HT1A receptor agonist radioligand [O-methyl-11C]2-(4-(4-(2-methoxyphenyl)piperazin-1-yl)butyl)-4-methyl-1,2,4-triazine-3,5(2H,4H)dione in nonhuman primates. *European Journal of Nuclear Medicine and Molecular Imaging* 34, 1050–1060. doi:10.1007/s00259-006-0324-y.

Lacivita, E., Niso, M., Hansen, H. D., Di Pilato, P., Herth, M. M., Lehel, S., et al. (2014). Design, synthesis, radiolabeling and in vivo evaluation of potential positron emission tomography (PET) radioligands for brain imaging of the 5-HT7 receptor. *Bioorganic & Medicinal Chemistry* 22, 1736–1750. doi:10.1016/j.bmc.2014.01.016.

Lemoine, L., Verdurand, M., Vacher, B., Blanc, E., Le Bars, D., Newman-Tancredi, A., et al. (2010). [18F]F15599, a novel 5-HT1A receptor agonist, as a radioligand for PET neuroimaging. *European Journal of Nuclear Medicine and Molecular Imaging* 37, 594–605. doi:10.1007/s00259-009-1274-y.

Lowe, P. T., Dall’Angelo, S., Mulder-Krieger, T., IJzerman, A. P., Zanda, M., and O’Hagan, D. (2017). A New Class of Fluorinated A2A Adenosine Receptor Agonist with Application to Last-Step Enzymatic [18 F]Fluorination for PET Imaging. *Chembiochem* 18, 2156–2164. doi:10.1002/cbic.201700382.

Majo, V. J., Milak, M. S., Prabhakaran, J., Mali, P., Savenkova, L., Simpson, N. R., et al. (2013). Synthesis and in vivo evaluation of [(18)F]2-(4-(4-(2-(2-fluoroethoxy)phenyl)piperazin-1-yl)butyl)-4-methyl-1,2,4-triazine-3,5(2H,4H)-dione ([(18)F]FECUMI-101) as an imaging probe for 5-HT1A receptor agonist in nonhuman primates. *Bioorg. Med. Chem.* 21, 5598–5604. doi:10.1016/j.bmc.2013.05.050.

Márián, T., Lehel, S., Lengyel, Z., Balkay, L., Horváth, G., Mikecz, P., et al. (2002). [The [18F]-FNECA serves as a suitable radioligand for PET investigation of purinergic receptor expression]. *Orv Hetil* 143, 1319–1322.

Marton, J., and Henriksen, G. (2012). Design and synthesis of an ^18^F-labeled version of phenylethyl orvinol ([^18^F]FE-PEO) for PET-imaging of opioid receptors. *Molecules* 17, 11554–11569. doi:10.3390/molecules171011554.

Marton, J., Schoultz, B., Hjo̷rnevik, T., Drzezga, A., Yousefi, B., Wester, H., et al. (2009). Synthesis and Evaluation of a Full-Agonist Orvinol for PET-Imaging. *C]PEO. Journal of Medicinal Chemistry* 52, 5586–5589. doi:doi:10.1021/jm900892x.

Mewshaw, R., Kavanagh, J., Stack, G., Marquis, K., Shi, X., Kagan, M., et al. (1997). New Generation Dopaminergic Agents. 1. Discovery of a Novel Scaffold Which Embraces the D 2 Agonist Pharmacophore. Structure−Activity Relationships of a Series of 2-(Aminomethyl)chromans. *J. Med. Chem* 40, 4235–4256. doi:doi:10.1021/jm9703653.

Milak, M. S., DeLorenzo, C., Zanderigo, F., Prabhakaran, J., Kumar, J. S. D., Majo, V. J., et al. (2010). In Vivo Quantification of Human Serotonin 1A Receptor Using 11C-CUMI-101, an Agonist PET Radiotracer. *Journal of Nuclear Medicine* 51, 1892–1900. doi:10.2967/jnumed.110.076257.

Milak, M. S., Severance, A. J., Ogden, R. T., Prabhakaran, J., Kumar, J. S. D., Majo, V. J., et al. (2008). Modeling Considerations for 11C-CUMI-101, an Agonist Radiotracer for Imaging Serotonin 1A Receptor In Vivo with PET. *Journal of Nuclear Medicine* 49, 587–596. doi:10.2967/jnumed.107.046540.

Milak, M. S., Severance, A. J., Prabhakaran, J., Kumar, J. D., Majo, V. J., Ogden, R. T., et al. (2011). *In vivo* Serotonin-Sensitive Binding of [ ^11^ C]CUMI-101: A Serotonin 1A Receptor Agonist Positron Emission Tomography Radiotracer. *Journal of Cerebral Blood Flow & Metabolism* 31, 243–249. doi:10.1038/jcbfm.2010.83.

Mogg, A. J., Eessalu, T., Johnson, M., Wright, R., Sanger, H. E., Xiao, H., et al. (2018). In Vitro Pharmacological Characterization and In Vivo Validation of LSN3172176 a Novel M1 Selective Muscarinic Receptor Agonist Tracer Molecule for Positron Emission Tomography. *J. Pharmacol. Exp. Ther.* 365, 602–613. doi:10.1124/jpet.117.246454.

Mukherjee, J., Majji, D., Kaur, J., Constantinescu, C. C., Narayanan, T. K., Shi, B., et al. (2017). PET radiotracer development for imaging high-affinity state of dopamine D2 and D3 receptors: Binding studies of fluorine-18 labeled aminotetralins in rodents: MUKHERJEE et al . *Synapse* 71, e21950. doi:10.1002/syn.21950.

Mukherjee, J., Narayanan, T., Christian, B., Shi, B., and and Yang, Z. (2004). Binding characteristics of high-affinity dopamine D2/D3 receptor agonists,11C-PPHT and11C-ZYY-339 in rodents and imaging in non-human primates by PET. *Synapse* 54, 83–91. doi:doi:10.1002/syn.20068.

Nabulsi, N. B., Holden, D., Zheng, M.-Q., Bois, F., Lin, S.-F., Najafzadeh, S., et al. (2019). Evaluation of ^11^ C-LSN3172176 as a novel PET tracer for imaging M _1_ muscarinic acetylcholine receptors in non-human primates. *Journal of Nuclear Medicine*, jnumed.118.222034. doi:10.2967/jnumed.118.222034.

Naik, R., Valentine, H., Dannals, R. F., Wong, D. F., and Horti, A. G. (2018). Synthesis and Evaluation of a New 18F-Labeled Radiotracer for Studying the GABAB Receptor in the Mouse Brain. *ACS Chem Neurosci* 9, 1453–1461. doi:10.1021/acschemneuro.8b00038.

Neelamegam, R., Hellenbrand, T., Schroeder, F. A., Wang, C., and Hooker, J. M. (2014). Imaging Evaluation of 5HT _2C_ Agonists, [ ^11^ C]WAY-163909 and [ ^11^ C]Vabicaserin, Formed by Pictet–Spengler Cyclization. *Journal of Medicinal Chemistry* 57, 1488–1494. doi:10.1021/jm401802f.

Palner, M., McCormick, P., Parkes, J., Knudsen, G., and and Wilson, A. (2010). Systemic catechol-O-methyl transferase inhibition enables the D1 agonist radiotracer R-[11C]SKF 82957. *Nuclear Medicine and Biology* 37, 837–843. doi:doi:10.1016/j.nucmedbio.2010.04.193.

Petersen, I. N., Villadsen, J., Hansen, H. D., Jensen, A. A., Lehel, S., Gillings, N., et al. (2016). Convergent 18 F-labeling and evaluation of N -benzyl-phenethylamines as 5-HT 2A receptor PET ligands. *Bioorganic & Medicinal Chemistry* 24, 5353–5356. doi:10.1016/j.bmc.2016.08.056.

Pinborg, L. H., Feng, L., Haahr, M. E., Gillings, N., Dyssegaard, A., Madsen, J., et al. (2012). No change in [11C]CUMI-101 binding to 5-HT1A receptors after intravenous citalopram in human. *Synapse* 66, 880–884. doi:10.1002/syn.21579.

Prabhakaran, J., Solingapuram Sai, K. K., Zanderigo, F., Rubin-Falcone, H., Jorgensen, M. J., Kaplan, J. R., et al. (2017). In vivo evaluation of [ 18 F]FECIMBI-36, an agonist 5-HT 2A/2C receptor PET radioligand in nonhuman primate. *Bioorganic & Medicinal Chemistry Letters* 27, 21–23. doi:10.1016/j.bmcl.2016.11.043.

Prabhakaran, J., Underwood, M. D., Kumar, J. S. D., Simpson, N. R., Kassir, S. A., Bakalian, M. J., et al. (2015). Synthesis and in vitro evaluation of [18F]FECIMBI-36: A potential agonist PET ligand for 5-HT2A/2C receptors. *Bioorganic & Medicinal Chemistry Letters* 25, 3933–3936. doi:10.1016/j.bmcl.2015.07.034.

Riss, P. J., Hong, Y. T., Marton, J., Caprioli, D., Williamson, D. J., Ferrari, V., et al. (2013). Synthesis and Evaluation of 18F-FE-PEO in Rodents: An 18F-Labeled Full Agonist for Opioid Receptor Imaging. *Journal of Nuclear Medicine* 54, 299–305. doi:10.2967/jnumed.112.108688.

Schou, M., Varnäs, K., Jucaite, A., Gulyás, B., Halldin, C., and Farde, L. (2013). Radiolabeling of the cannabinoid receptor agonist AZD1940 with carbon-11 and PET microdosing in non-human primate. *Nucl. Med. Biol.* 40, 410–414. doi:10.1016/j.nucmedbio.2012.10.011.

Selvaraj, S., Mouchlianitis, E., Faulkner, P., Turkheimer, F., Cowen, P. J., Roiser, J. P., et al. (2015). Presynaptic Serotoninergic Regulation of Emotional Processing: A Multimodal Brain Imaging Study. *Biol. Psychiatry* 78, 563–571. doi:10.1016/j.biopsych.2014.04.011.

Selvaraj, S., Turkheimer, F., Rosso, L., Faulkner, P., Mouchlianitis, E., Roiser, J. P., et al. (2012). Measuring endogenous changes in serotonergic neurotransmission in humans: a [11C]CUMI-101 PET challenge study. *Molecular Psychiatry* 17, 1254–1260. doi:10.1038/mp.2012.78.

Selvaraj, S., Walker, C., Arnone, D., Cao, B., Faulkner, P., Cowen, P. J., et al. (2018). Effect of Citalopram on Emotion Processing in Humans: A Combined 5-HT1A [11C]CUMI-101 PET and Functional MRI Study. *Neuropsychopharmacology* 43, 655–664. doi:10.1038/npp.2017.166.

Shalgunov, V., van Wieringen, J.-P., Janssen, H. M., Fransen, P. M., Dierckx, R. A. J. O., Michel, M. C., et al. (2015). Synthesis and evaluation in rats of homologous series of [18F]-labeled dopamine D2/3 receptor agonists based on the 2-aminomethylchroman scaffold as potential PET tracers. *EJNMMI Research* 5. doi:10.1186/s13550-015-0119-x.

Shi, B., Narayanan, T., Christian, B., Chattopadhyay, S., and and Mukherjee, J. (2004). Synthesis and biological evaluation of the binding of dopamine D2/D3 receptor agonist, (R,S)-5-hydroxy-2-(N-propyl-N-(5′-18F-fluoropentyl)aminotetralin (18F-5-OH-FPPAT) in rodents and nonhuman primates. *Nuclear Medicine and Biology* 31, 303–311. doi:doi:10.1016/j.nucmedbio.2003.10.004.

Shi, B., Narayanan, T., Yang, Z., Christian, B., and and Mukherjee, J. (1999). Radiosynthesis and in vitro evaluation of 2-(N-alkyl-N-1′-11C-propyl)amino-5-hydroxytetralin analogs as high affinity agonists for dopamine D-2 receptors. *Nuclear Medicine and Biology* 26, 725–735. doi:doi:10.1016/s0969-8051(99)00051-7.

Shiue, C.-Y., Bai, L.-Q., Teng, R.-R., Arnett, C. D., Dewey, S. L., Wolf, A. P., et al. (1991). A comparison of the brain uptake of N-(cyclopropyl[11C]methyl)norbuprenorphine ([11C]buprenorphine) and N-(cyclopropyl[11C]methyl)nordiprenorphme ([11C]diprenorphine) in baboon using PET. *International Journal of Radiation Applications and Instrumentation. Part B. Nuclear Medicine and Biology* 18, 281–288. doi:10.1016/0883-2897(91)90123-3.

Shrestha, S. S., Liow, J.-S., Jenko, K., Ikawa, M., Zoghbi, S. S., and Innis, R. B. (2016). The 5-HT1A Receptor PET Radioligand 11C-CUMI-101 Has Significant Binding to α1-Adrenoceptors in Human Cerebellum, Limiting Its Use as a Reference Region. *J. Nucl. Med.* 57, 1945–1948. doi:10.2967/jnumed.116.174151.

Shrestha, S. S., Liow, J.-S., Lu, S., Jenko, K., Gladding, R. L., Svenningsson, P., et al. (2014). 11C-CUMI-101, a PET Radioligand, Behaves as a Serotonin 1A Receptor Antagonist and Also Binds to 1 Adrenoceptors in Brain. *Journal of Nuclear Medicine* 55, 141–146. doi:10.2967/jnumed.113.125831.

Sromek, A., Si, Y., Zhang, T., George, S., Seeman, P., and and Neumeyer, J. (2011). Synthesis and Evaluation of Fluorinated Aporphines: Potential Positron Emission Tomography Ligands for D 2 Receptors. ACS Med. *Chem. Lett* 2, 189–194. doi:doi:10.1021/ml1001689.

Terry, G. E., Hirvonen, J., Liow, J.-S., Zoghbi, S. S., Gladding, R., Tauscher, J. T., et al. (2010). Imaging and Quantitation of Cannabinoid CB1 Receptors in Human and Monkey Brains Using 18F-Labeled Inverse Agonist Radioligands. *Journal of Nuclear Medicine* 51, 112–120. doi:10.2967/jnumed.109.067074.

Terry, G., Liow, J., Chernet, E., Zoghbi, S., Phebus, L., Felder, C., et al. (2008). Positron emission tomography imaging using an inverse agonist radioligand to assess cannabinoid CB1 receptors in rodents. *NeuroImage* 41, 690–698. doi:doi:10.1016/j.neuroimage.2008.03.004.

Terry, G., Liow, J., Zoghbi, S., Hirvonen, J., Farris, A., Lerner, A., et al. (2009). Quantitation of cannabinoid CB1 receptors in healthy human brain using positron emission tomography and an inverse agonist radioligand. *NeuroImage* 48, 362–370. doi:doi:10.1016/j.neuroimage.2009.06.059.

Tsujikawa, T., Zoghbi, S., Hong, J., Donohue, S., Jenko, K., Gladding, R., et al. (2014). In vitro and in vivo evaluation of 11C-SD5024, a novel PET radioligand for human brain imaging of cannabinoid CB1 receptors. *NeuroImage* 84, 733–741. doi:doi:10.1016/j.neuroimage.2013.09.043.

van Wieringen, J.-P., Shalgunov, V., Janssen, H. M., Fransen, P. M., Janssen, A. G. M., Michel, M. C., et al. (2014). Synthesis and Characterization of a Novel Series of Agonist Compounds as Potential Radiopharmaceuticals for Imaging Dopamine D _2/3_ Receptors in Their High-Affinity State. *Journal of Medicinal Chemistry* 57, 391–410. doi:10.1021/jm401384w.

Vangveravong, S., Zhang, Z., Taylor, M., Bearden, M., Xu, J., Cui, J., et al. (2011). Synthesis and characterization of selective dopamine D2 receptor ligands using aripiprazole as the lead compound. *Bioorganic & Medicinal Chemistry* 19, 3502–3511. doi:doi:10.1016/j.bmc.2011.04.021.

Xu, J., Vangveravong, S., Li, S., Fan, J., Jones, L., Cui, J., et al. (2013). Positron emission tomography imaging of dopamine D2 receptors using a highly selective radiolabeled D2 receptor partial agonist. *NeuroImage* 71, 168–174. doi:doi:10.1016/j.neuroimage.2013.01.007.

Yasuno, F., Brown, A. K., Zoghbi, S. S., Krushinski, J. H., Chernet, E., Tauscher, J., et al. (2008). The PET Radioligand [11C]MePPEP Binds Reversibly and with High Specific Signal to Cannabinoid CB1 Receptors in Nonhuman Primate Brain. *Neuropsychopharmacology* 33, 259–269. doi:10.1038/sj.npp.1301402.
